# Sequence Analysis of Insecticide Action and Detoxification-Related Genes in the Insect Pest Natural Enemy *Pardosa pseudoannulata*

**DOI:** 10.1371/journal.pone.0125242

**Published:** 2015-04-29

**Authors:** Xiangkun Meng, Yixi Zhang, Haibo Bao, Zewen Liu

**Affiliations:** 1 Key Laboratory of Integrated Management of Crop Diseases and Pests (Ministry of Education), College of Plant Protection, Nanjing Agricultural University, Nanjing 210095, China; 2 Institute of Plant Protection, Jiangsu academy of agricultural sciences, Nanjing 210014, China; Institute of Zoology, Chinese Academy of Sciences, CHINA

## Abstract

The pond wolf spider *Pardosa pseudoannulata*, an important natural predatory enemy of rice planthoppers, is found widely distributed in paddy fields. However, data on the genes involved in insecticide action, detoxification, and response are very limited for *P*. *pseudoannulata*, which inhibits the development and appropriate use of selective insecticides to control insect pests on rice. We used transcriptome construction from adult spider cephalothoraxes to analyze and manually identify genes enconding metabolic enzymes and target receptors related to insecticide action and detoxification, including 90 cytochrome P450s, 14 glutathione *S*-transferases (GSTs), 17 acetylcholinesterases (AChEs), 17 nicotinic acetylcholine receptors (nAChRs), and 17 gamma-aminobutyric acid (GABA) receptors, as well as 12 glutamate-gated chloride channel (GluCl) unigenes. Sequence alignment and phylogenetic analysis revealed the different subclassifications of P450s and GSTs, some important sequence diversities in nAChRs and GABA receptors, polymorphism in AChEs, and high similarities in GluCls. For P450s in *P*. *pseudoannulata*, the number of unigenes belonging to the CYP2 clade was much higher than that in CYP3 and CYP4 clades. The results differed from insects in which most P450 genes were in CYP3 and CYP4 clades. For GSTs, most unigenes belonged to the delta and sigma classes, and no epsilon GST class gene was found, which differed from the findings for insects and acarina. Our results will be useful for studies on insecticide action, selectivity, and detoxification in the spider and other related animals, and the sequence differences in target genes between the spider and insects will provide important information for the design of selective insecticides.

## Introduction

Biological control is an important way for pest management. Currently, increasing interest in safe, effective, and sustainable strategies for insect pest control is encouraging the use of biological control strategies, but the primary way for insect pests control in China remains the application of chemical insecticides, which not only cause environmental contamination but also reduce the populations of natural enemies [1]. Spiders are recognized as important natural enemies to reduce pest populations. The pond wolf spider *Pardosa pseudoannulata*, an important predatory enemy of rice planthoppers and leafhoppers, is one of the most abundant spiders in paddy fields and able to effectively reduce rice pest populations. Nevertheless, spiders have been variably affected by the extensive use of insecticides, especially neurotoxic substances [2]. Neonicotinoid insecticides, acting selectively as neurotoxins on insect nicotinic acetylcholine (ACh) receptors (nAChRs) and extensively used to control rice insect pests, demonstrated relative safety to *P*. *pseudoannulata* [3]. In previous studies, the chemical structure and differences in the nAChRs between insects and *P*. *pseudoannulata* were found to contribute directly to neonicotinoid sensitivities [4–6].

To develop approaches to protect the natural enemy *P*. *pseudoannulata* and improve insecticide selectivity, pharmacological studies of insects and *P*. *pseudoannulata* are necessary. Molecular mechanisms of insecticide resistance in insects have been studied in depth, but such studies on spiders are rare, with only 3% of the toxicology papers on natural enemies devoted to spiders [2]. Two principal regulatory molecular mechanisms of insecticide resistance exist in insects: enhanced insecticide metabolism and reduced sensitivity of targets to insecticides [7]. Enhanced insecticide metabolism is mainly due to the increased metabolism enzyme activities or overexpression of enzyme genes, such as cytochrome P450s and glutathione *S-*transferases (GSTs) [8]. Reduced sensitivity of targets to insecticides mainly occurs through target mutation by reducing binding of the insecticide to its target [9]. The most frequently studied targets are acetylcholinesterases (AChEs), nicotinic acetylcholine receptors (nAChRs), gamma-aminobutyric acid (GABA) receptors, and glutamate-gated chloride channels (GluCls).

The lack of genetic information on *P*. *pseudoannulata* limits the studies of differences between insects and *P*. *pseudoannulata*, especially the genes related to insecticide action and detoxification. Prior to this study, only 11 amino acid sequences of *P*. *pseudoannulata* were found in NCBI (National Center for Biotechnology Information) database (http://www.ncbi.nlm.nih.gov), including four nAChR subunits and two AChE genes. Fortunately, high-throughput sequencing technology can provide the rapid high-quality genetic information. In the present study, we used the high-throughput sequencing Illumina HiSeq 2000 instrument to complete sequencing of the entire transcriptome and acquired a large volume of genetic information on *P*. *pseudoannulata*. Genes related to insecticide action and detoxification were manually identified and analyzed in sequences.

## Results and Discussion

### De novo assembly, sequence homology distribution, and function annotations

Using Illumina sequencing, *P*. *pseudoannulata* transcriptome was generated from male and female adult spider cephalothoraxes. Basic information on the transcriptome is summarized in S1 Table. Length distribution and homology analysis of unigenes that hit in the NCBI nonredundant (Nr) protein database are shown in S1 and S2 Figs To acquire accurate annotation information, the Gene Ontology (GO), Clusters of Orthologous Groups (COG), and Kyoto Encyclopedia of Genes and Genomes (KEGG) databases were also used to annotate the unigenes (S3 and S4 Figs, and S2 Table). The transcriptome data have been deposited in NCBI (accession number: GCKE00000000).

### Identification and analysis of genes related to insecticide action and detoxification

Insecticides such as neonicotinoids show high toxicity to insect pests, but relative hypotoxicity to mammal and natural enemies. One of the main reasons for this is the differences in their insecticide targets and metabolism enzymes [6, 10]. As an important predatory enemy of rice insect pests, genetic information on *P*. *pseudoannulata* is highly lacking. In this study, the *P*. *pseudoannulata* transcriptome supplied significantly more genetic information than that from the literatures. The genes related to insecticide action and detoxification were manually identified, including the metabolism enzymes cytochrome P450s and GSTs, and insecticide targets AChEs, nAChRs, GABA receptors, and GluCls (Table 1).

**Table 1:** Statistical data for unigenes hit in the NCBI nonredundant (Nr) database associated with insecticide action and detoxification in the *P*. *pseudoannulata* transcriptome.

Unigene category	Unigene number	Maximum unigene length	Minimum unigene length	Average length
P450s	90	1885	150	583
nAChRs	17	1104	150	478
AChEs	17	2853	153	1116
VGSCs	13	5359	159	919
GSTs	14	965	384	750
GluCls	12	1786	221	955
GABA receptors	17	1943	150	714
Cyt b	27	1700	153	588
ATPase	111	4911	150	913
CarEs	3	933	170	447
Hsp	31	3012	160	1115
RyRs	6	8445	156	3662
Cl^-^ channel	15	3213	160	807

### Cytochrome P450 family

**Fig 1:**
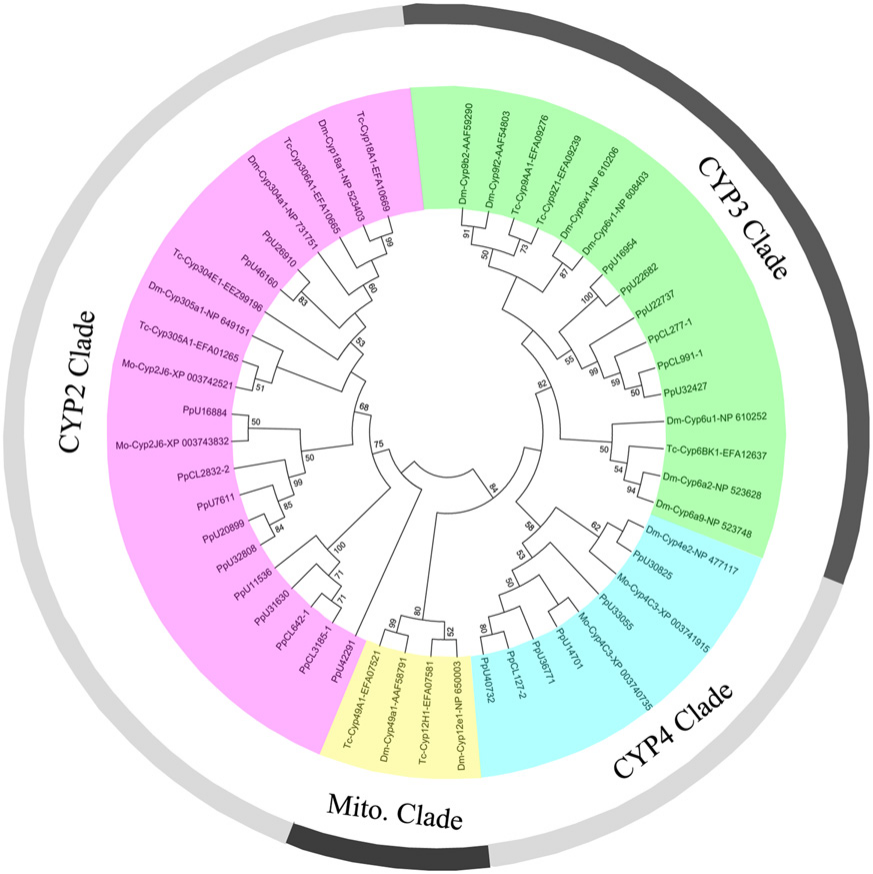
Phylogenetic analysis of putative cytochrome P450 genes in *P*. *pseudoannulata* compared with insect P450s. Numbers above the branches indicate phylogenies from amino acid sequences and only values above 50% are shown. Dm: *Drosophila melanogaster*; Tc: *Tribolium castaneum*; Mo: *Metaseiulus occidentalis*.

The cytochrome P450 family, one of the largest superfamilies of insect species, has functions in xenobiotic metabolism and detoxification. The P450 superfamily includes four main clades: CYP2, CYP3, CYP4, and the mitochondrial CYP clade (CYP M) [11]. The multitudinous and highly active nature of P450s allows insects to metabolize nearly all classes of insecticides and other xenobiotics [12]. Changes in P450s were found to result in high insecticide resistance in many insect species [13–21]. In our dataset, 90 P450 unigenes with an average length of 583 bp were hit in the NCBI Nr database, and 79 putative P450 genes were identified (S3 Table). Most P450 unigenes belonged to CYP2 clade (34/90), followed by CYP4 and CYP3 clades (19/90 and 17/90), and nine unigenes were annotated to CYP M (9/90). However, due to the existence of unigenes with a short open reading frame and fragmentary distribution, only 24 P450 unigenes were well classified in the sequence alignment and phylogenetic analysis with model insect P450s (Fig 1).

In insects, the CYP3 clade contains the most numerous P450 genes and functions in xenobiotic metabolism and insecticide resistance. Studies have found that changes in allele or overexpression of genes in CYP3 clade resulted in high insecticide resistance in insects, such as CYP337B3 and CYP6AY1 [17, 21]. The CYP4 clade P450 genes in insects were also found to be involved in both pesticide metabolism and chemical communication [22]. Overexpression of genes in the CYP4 clade also could lead to high insecticide resistance, e.g., CYP4C27 and CYP4G19 [23, 24]. The mitochondrial P450s are all involved in steroid or vitamin D metabolism in vertebrates and xenobiotic metabolism in insects [25]. CYP2 clade is a relatively small clade when compared to CYP3 and CYP4 clades in insects, with the primary functions involved in juvenile hormone biosynthesis and ecdysone metabolism. In the present *P*. *pseudoannulata* transcriptome, CYP2 clade was superior in P450 unigene number and was close to the sum of CYP3 and CYP4 clade. This result differed markedly from that for insects, in which more P450 genes were found in CYP3 and CYP4 clades [26–30]. These findings indicated that the significant differences existed in xenophobic metabolism and detoxification between arachnoidea and insects.

### Glutathione *S-*transferase

GST (E.C. 2.5.1.18) belongs to the multifunctional detoxification enzymes involved in the detoxification of xenobiotics. GSTs catalyze the conjugation of reduced glutathione (GSH) with exogenous and endogenous toxic compounds or their metabolites, rendering them more water-soluble, less toxic, and easier to excrete [31]. In insects, increased expression and/or activity of GSTs can enhance insecticide resistances [32, 33]. GSTs consist of seven main classes: delta, epsilon, omega, sigma, theta, zeta, and microsomal. The delta and epsilon classes have been described as the insect-specific GST classes and implicated in xenobiotic metabolism, especially in insecticide detoxification. An epsilon GST was able to detoxify DDT and result in DDT-resistance in *Anopheles gambiae* [8]. Sigma GSTs show low activity with the typical GST substrates, but have high affinity with the lipid peroxidation product, 4-hydroxynonenal, and are localized in metabolically active tissues in flies [22]. Like the cytosolic enzymes, the microsomal GSTs play a similar role in general detoxification reactions and protection against oxidative stress [22].

**Fig 2:**
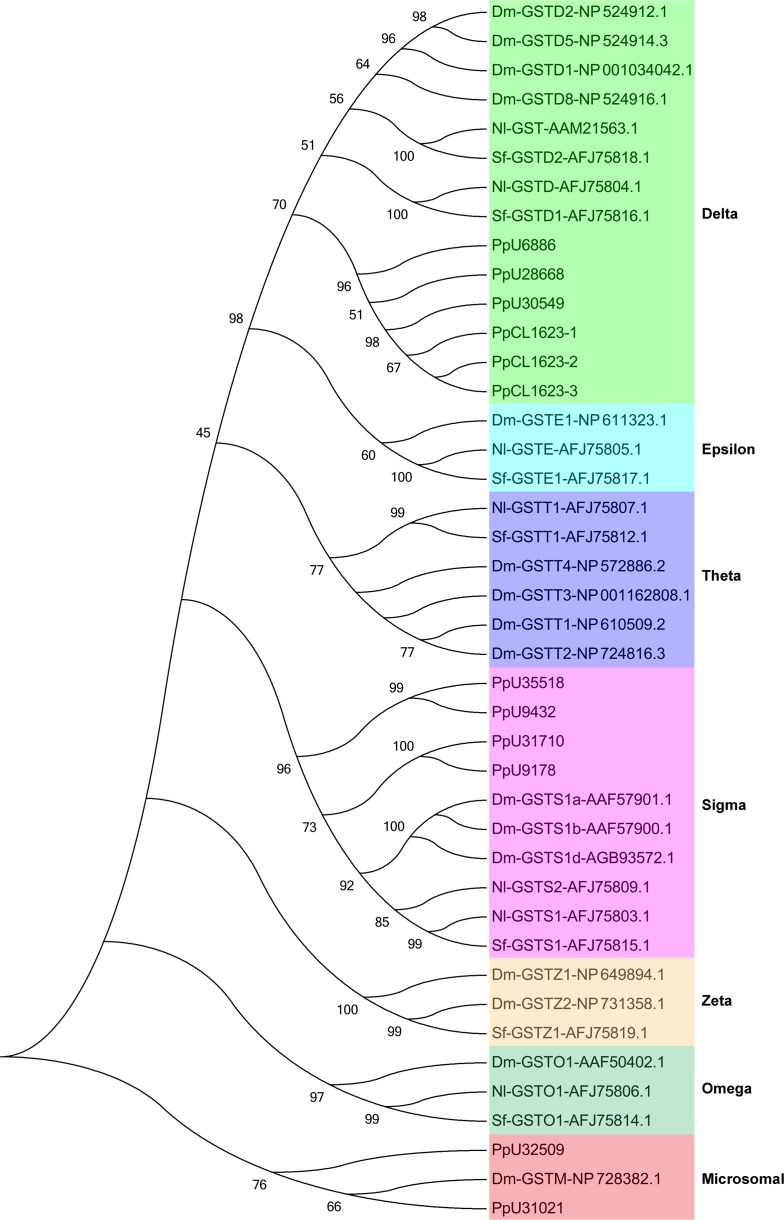
Phylogenetic analysis of GST unigenes in the *P*. *pseudoannulata* transcriptome compared with insect GSTs. Numbers above the branches indicate phylogenies from amino acid sequences and only values above 45% are shown. Dm: *Drosophila melanogaster*; Nl: *Nilaparvata lugens*; Sf: *Sogatella furcifera*.

In the present study, 14 GST unigenes were found in the *P*. *pseudoannulata* transcriptome belonging to three classes: delta (6/14), sigma (5/14), microsomal (2/14), and one unknown (S4 Table). Phylogenetic analysis with model insects produced 12 GST unigenes (Fig 2), most of which belonged to the delta and sigma classes, and no epsilon GST gene was found. The results differed from those for insects, in which the main GST classes were delta and epsilon, with no microsomal class. Moreover, the findings were also different from the main GST classes in acarina, which were delta and microsomal classes, but without sigma class [34]. In the multiped spider *P*. *pseudoannulata*, which has no wings, the sigma class GSTs possibly mainly function in the muscles related to jumping, but not in flight muscles.

### Acetylcholinesterase

**Fig 3:**
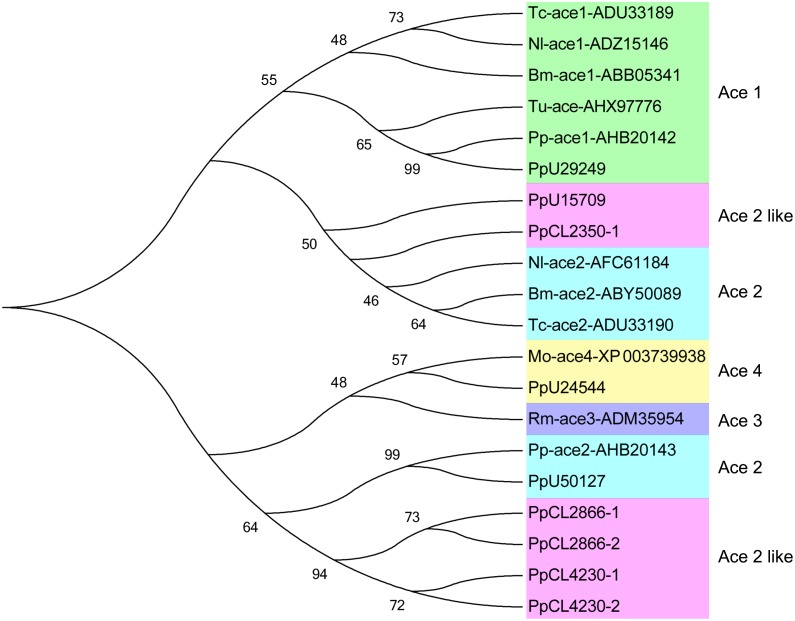
Phylogenetic analysis of AChE unigenes in the *P*. *pseudoannulata* transcriptome compared with other species’ AChEs. Numbers above the branches indicate phylogenies from amino acid sequences and only values above 45% are shown. Tc: *Tribolium castaneum*; Nl: *Nilaparvata lugens*; Bm: *Bombyx mori*; Tu: *Tetranychus urticae*; Rm: *Rhipicephalus microplus*; Mo: *Metaseiulus occidentalis*; Pp: *Pardosa pseudoannulata*.

AChE (EC 3.1.1.7) is an important neurotransmitter hydrolase in insect nervous system that terminates transmission at the cholinergic synapses by hydrolyzing ACh [35]. Organophosphate and carbamate insecticides act as inhibitors on AChEs, blocking normal nerve conduction and finally resulting in the death of insects. Studies have found that changes in insect AChEs could lead to high resistance to many insecticides. In several insects, such as *Drosophila melanogaster*, *Leptinotarsa decemlineata*, *Nilaparvata lugens*, and *Plutella xylostella*, a single amino acid mutation could decrease the sensitivity of AChEs to a single insecticide alone or insecticides used in combination. [36–39]. In our previous study, two AChE genes cloned from *P*. *pseudoannulata* exhibited significantly different sensitivities to insecticides, and some key amino acid differences between *P*. *pseudoannulata* and insect AChEs might play important roles in insecticide selectivity [35]. In the present study, 17 AChE unigenes were identified from the *P*. *pseudoannulata* transcriptome (S5 Table). Among the unigenes, two were perfectly hit to previously reported AChE1 and AChE2, and one unigene showed high similarity to *Metaseiulus occidentalis* AChE4 (Fig 3). However, several unigenes also showed high similarity to *P*. *pseudoannulata* AChE2, such as contig2866, contig4230, contig2350, and unigene15709 (Fig 3). The results indicated that *P*. *pseudoannulata* may have more than four AChEs. The number of ace genes in invertebrates varies, e.g., four in nematodes, one or two in different insect species, and one in the spider mite *Tetranychus urticae* [35]. Further studies are required to determine the number and function of AChEs in *P*. *pseudoannulata*.

### Nicotinic acetylcholine receptors

**Fig 4:**
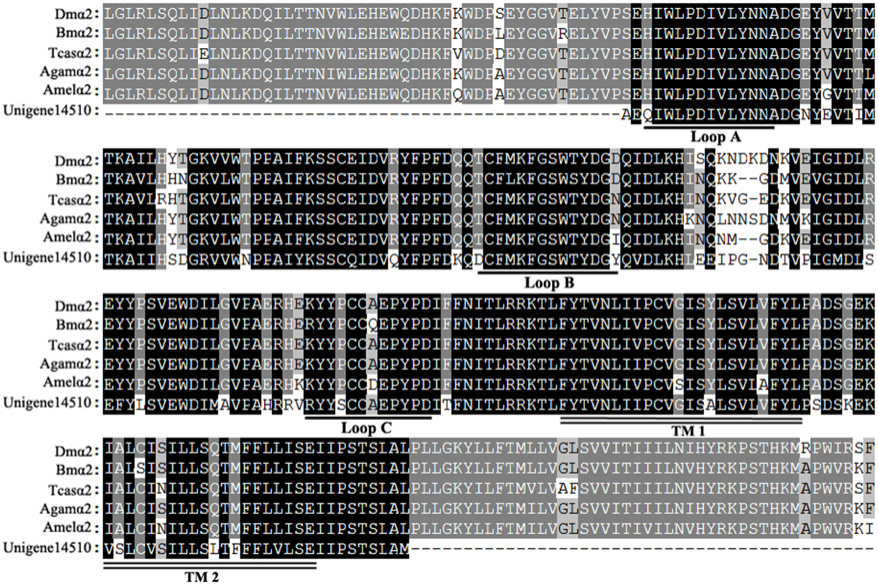
Alignment of unigene14510 in the *P*. *pseudoannulata* transcriptome with insect α2 subunits. Loops A, B, and C, important in agonist binding in the nAChR α subunit, are underscored by a single line. Transmembrane domains (TM 1–2) are marked by double lines. Dm: *Drosophila melanogaster* (NP_524482); Bm: *Bombyx mori* (ABV72684); Tcas: *Tribolium castaneum* (EFA10793); Aga: *Anopheles gambiae* (AAU12504); Amel: *Apis mellifera* (NP_001011625).

nAChRs are ligand-gated ion channels and abundant within the nervous systems of both invertebrates and vertebrates, mediating fast cholinergic synaptic transmission. They are targets of many insecticides, such as neonicotinoids, nereistoxin, and spinosad [4]. In studies of nAChRs, ~10–12 nAChR subunits were found in insects. Previous studies showed that residue mutations in a key region of nAChR subunits influenced insecticide sensitivity, and in many cases represented important mechanisms for insecticide resistance [40–44]. Neonicotinoids selectively act on insect nAChRs, but show relative safety for mammals and *P*. *pseudoannulata*, partly because of the differences in nAChRs [5]. In our previous study on nAChRs, key amino acid differences between *P*. *pseudoannulata* and insect β1 influenced neonicotinoid sensitivity [45]. In the present transcriptome, 17 nAChR unigenes were identified, including six alpha subunits (α2, α3, α6, α7, α8, α9) and two beta subunits (β2, β3) (S6 Table, S5 Fig). The amino acid differences were found in key regions in specific subunits between *P*. *pseudoannulata* and insects. For example, differences were found in loops A, B, and C of α2 and α6 subunits between *P*. *pseudoannulata* and insects (Fig 4 and S5 Fig).

### Ionotropic γ-aminobutyric acid receptor

Insect ionotropic γ-aminobutyric acid (GABA_A_) receptor is a chloride channel and an important target for insecticides such as fipronil and cyclodienes, which act as antagonists and bind with higher affinities to insect than to vertebrate ionotropic GABA_A_ receptors [46]. In insects, the GABA_A_ receptor has three subunits: Rdl (resistance to dieldrin), Lcch3 (ligand-gated chloride channel homolog 3), and Grd (the GABA and glycine-like receptor of *Drosophila*). Studies have found that amino acid substitution in Rdl could confer insecticide resistance; e.g., A302S and A302G resulted in dieldrin resistance in *D*. *melanogaster* and *D*. *simulans*, A296S substitution was associated with dieldrin resistance in *A*. *gambiae*, *Anopheles arabiensis*, and *Aedes aegypti*, and an A302N mutation conferred resistance to fipronil in *Sogatella furcifera* [47–49].

**Fig 5:**
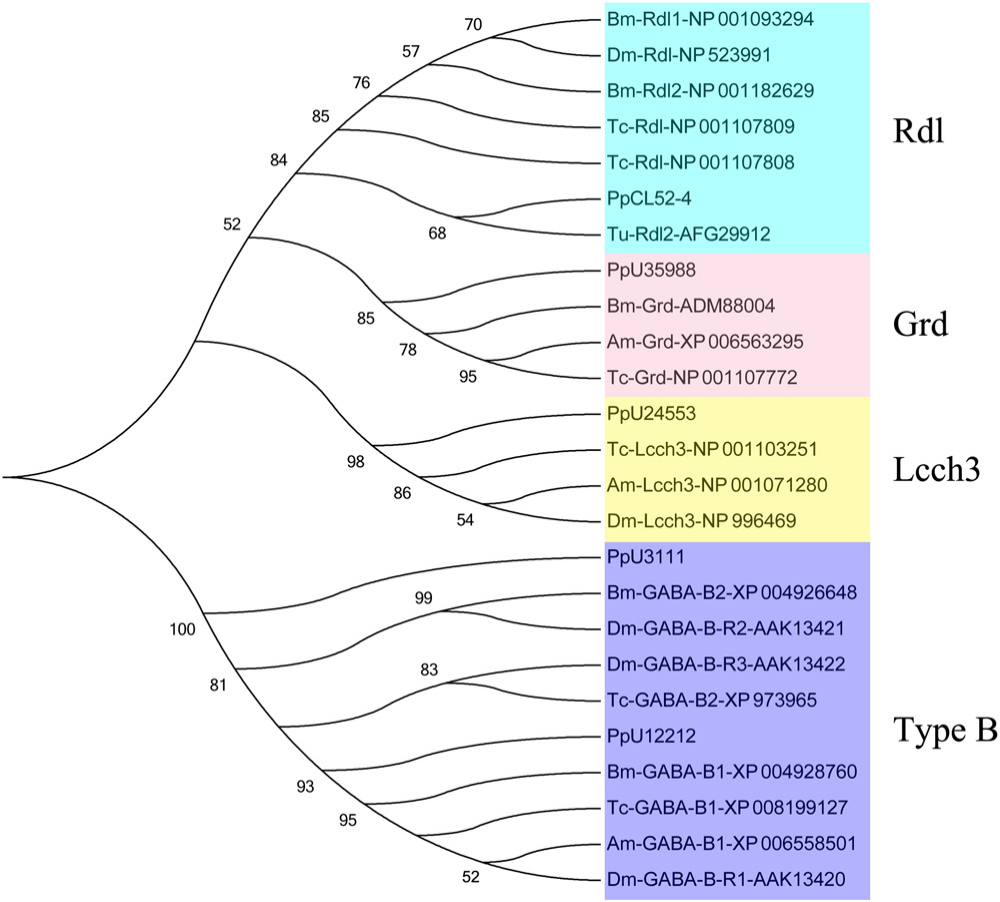
Phylogenetic analysis of GABA receptor unigenes in the *P*. *pseudoannulata* transcriptome with the GABA receptors of other species. Numbers above the branches indicate phylogenies from amino acid sequences and only values above 50% are shown. Bm: *Bombyx mori*; Dm: *Drosophila melanogaster*; Tc: *Tribolium castaneum*; Tu: *Tetranychus urticae*; Am: *Apis mellifera*.

In the present transcriptome, 17 GABA receptor unigenes were found, including nine GABA_A_ receptor unigenes and eight GABA_B_ receptor unigenes (S7 Table). Four Rdl genes, three Lcch3 genes, and one Grd gene were identified using phylogenetic analysis and NCBI Nr annotation in GABA_A_ receptor unigenes (Fig 5). Some of the annotated Rdl, Lcch3, and Grd unigenes were manually aligned with GABA_A_ receptor genes from other species and showed very high similarities (S6 Fig). The Rdl gene is attracting significant research interest in the field of insecticide resistance, but it has not been reported in the literature for the natural enemy spider. In the present study, we acquired almost the complete sequence of Rdl for *P*. *pseudoannulata*, which could aid in analyzing the differences between insects and natural enemies.

### Glutamate-gated chloride channel

**Fig 6:**
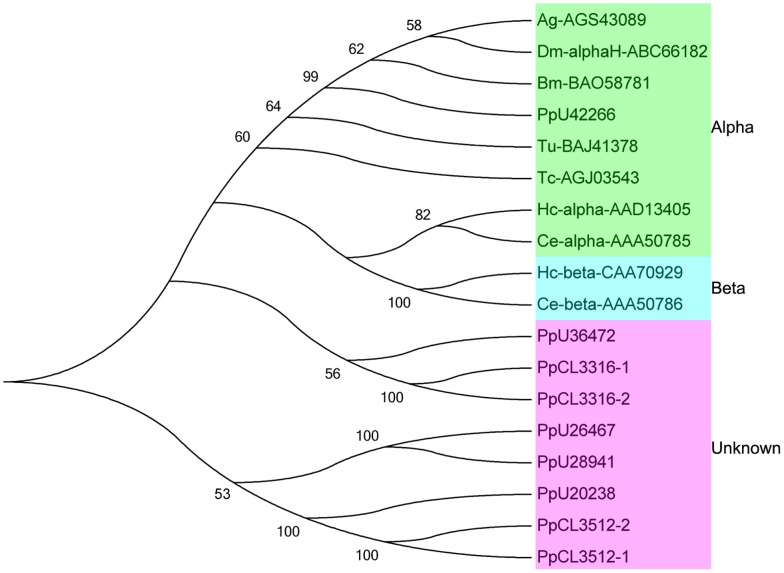
Phylogenetic analysis of GluCl unigenes in the *P*. *pseudoannulata* transcriptome with other species’ GluCls. Numbers above the branches indicate phylogenies from amino acid sequences and only values above 50% are shown. Ag: *Anopheles gambiae*; Dm: *Drosophila melanogaster*; Bm: *Bombyx mori*; Tu; *Tetranychus urticae*; Tc: *Tetranychus cinnabarinus*; Hc: *Haemonchus contortus*; Ce: *Caenorhabditis elegans*.

GluCls, as members of the cysteine loop ligand-gated ion channel superfamily, are important targets for various insecticides, such as avermectin, ivermectin, and fipronil. To date, GluCls are only found in invertebrates, such as insects, which make them the ideal targets for insecticides [50]. However, insecticide resistance is a major problem in insecticides targeting GluCls. Studies have shown that mutations or alternative splicing in GluCl subunits can reduce insecticide sensitivities [51, 52]. In the present study, 12 GluCls unigenes were found with an average length of 955 bp (S8 Table). Phylogenetic analysis and sequence alignment were also employed in related unigenes, and several unigenes showed high similarities with insect, nematode, and acarid GluCls (Fig 6). Notably, the transmembrane regions of unigene42266 were mostly identical to insect GluCls (S7 Fig). The high similarity in GluCls between *P*. *pseudoannulata* and insects suggests that *P*. *pseudoannulata* may also show high sensitivity to insecticides targeting insect GluCls.

## Conclusions

The transcriptome provided significant genetic information on the natural predator *P*. *pseudoannulata*. The genes related to insecticide action and detoxification were manually identified, including 90 P450s, 14 GSTs, 17 AChEs, 17 nAChRs, 17 GABA receptors, and 12 GluCl unigenes. The main categories and differences in these genes between insects and *P*. *pseudoannulata* were analyzed, which provide useful information for the development of selective insecticides between targeted insect pests and this natural enemy.

## Materials and Methods

### Sample preparation

*P*. *pseudoannulata* was collected from a field of hybrid paddy rice in Nanjing (Jiangsu, China). Spiders were narcotized with CO_2_ and the legs, chelicerae, and abdomen were manually removed. Samples contained an equal ratio of male and female adult spiders. We confirmed that the location was not privately owned or protected in any way and that the species collections did not involve endangered or protected species.

### RNA isolation, library construction, and Illumina sequencing

Total RNA was isolated using TRIzol reagent (Invitrogen/Life Technologies, Paisley, UK). RNA purity and integrity were assessed by the absorbance ratio and agarose gel electrophoresis, and the qualified RNA sample was used for cDNA library construction. mRNA was enriched, fragmented, and used as the template to synthesize the first strand cDNA, after which the second strand cDNA was synthesized. These cDNA fragments were purified and resolved with buffer EB (Elution Buffer) for end reparation and addition of poly(A), then ligated to adaptors. Suitable fragments were selected as templates for PCR amplification to create the cDNA library. Finally, the cDNA library was sequenced using the Illumina HiSeq 2000 (Illumina Inc., San Diego, CA, USA).

### De novo assembly and transcript annotation

Before assembly, low-quality reads were removed from the raw reads to generate clean reads. De novo transcriptome analysis of the clean reads were assembled using the short-read assembly program Trinity [53].

Finally, all assembled unigenes with a significant cutoff E-value <10^–5^ were determined using BLASTx against the NCBI Nr protein, Swiss-Prot, KEGG, and the COG databases. The best matches were used to identify coding regions and to determine the sequence direction. If the alignment results of different databases conflicted with each other, a priority order of NCBI Nr, Swiss-Prot, KEGG, and COG was used. The NCBI Nr database was used to predict the functional annotations of sequences, the GO annotation of unigenes using Blast2GO and WEGO to perform GO functional classification, and to determine the distribution of the gene functions of the species [54]. The KEGG database was used to analyze gene products during metabolism processes and related gene functions in cellular processes [34]. The unigene sequences were also aligned to the COG database to predict and classify functions [55].

### Identification and analysis of genes related to insecticide action and detoxification

Genes related to insecticide action and detoxification were identified from the BLAST results generated with the NCBI Nr database with a cutoff E-value of <10^–5^. The unigenes found in the same BLAST results or with high homology to one another were eliminated selectively as allelic variants or as different parts of the same gene [26]. The complete coding region was determined using the open reading frame finder (http://www.ncbi.nlm.nih.gov/gorf/gorf.html), and protein BLAST results were generated. Genes of other species were downloaded from the NCBI database and used as references. MEGA 5.05 software was used to analyze the phylogenetic relationships between genes of interest with the related genes of other species. Phylogenetic trees were generated using the neighbor-joining method and bootstrapped with 1000 iterations to evaluate the branch strength of the tree [56, 57].

## Supporting Information

S1 Fig Length distribution of *P*. *pseudoannulata* transcriptome sequences. (DOCX)

S2 Fig Homology analysis of unigenes for *P*. *pseudoannulata*. (DOCX)

S3 Fig Gene Ontology (GO) annotation and classification of the *P*. *pseudoannulata* transcriptome. (DOCX)

S4 Fig Clusters of orthologous group (COG) function classification of the *P*. *pseudoannulata* transcriptome. (DOCX)

S5 Fig Alignment of insect nAChR subunits with unigenes in the *P*. *pseudoannulata* transcriptome. (DOCX)

S6 Fig Alignment of species GABA receptors, Rdl, Lcch3, and Grd, with unigenes in the *P*. *pseudoannulata* transcriptome. (DOCX)

S7 Fig Alignment of species glutamate-gated chloride channels with unigenes in the *P*. *pseudoannulata* transcriptome. (DOCX)

S1 Table Summary of the statistics for Illumina sequencing of the *P*. *pseudoannulata* transcriptome. (DOCX)

S2 Table Distribution of KEGG function classification of the *P*. *pseudoannulata* transcriptome. (XLSX)

S3 Table Manually identified P450 unigenes from the *P*. *pseudoannulata* transcriptome. (DOCX)

S4 Table Manually identified GST unigenes from the *P*. *pseudoannulata* transcriptome. (DOCX)

S5 Table Manually identified AChE unigenes from the *P*. *pseudoannulata* transcriptome. (DOCX)

S6 Table Manually identified nAChR unigenes from the *P*. *pseudoannulata* transcriptome. (DOCX)

S7 Table Manually identified GABA receptor unigenes from the *P*. *pseudoannulata* transcriptome. (DOCX)

S8 Table Manually identified GluCl unigenes from the *P*. *pseudoannulata* transcriptome. (DOCX)

## References

[1] Lou YG, Zhang GR, Zhang WQ, Hu Y, Zhang J. Reprint of: Biological control of rice insect pests in China. Biological Control. 2014;68: 103–116. 10.1016/j.biocontrol.2013.09.018 24062411

[2] Pekar S. Spiders (Araneae) in the pesticide world: an ecotoxicological review. Pest Management Science. 2012;68(11): 1438–1446. 10.1002/ps.3397 . 22945871

[3] Tang LD, Qiu BL, Ren SX. A review of insecticide resistance in the natural enemies of pest insects. Chinese Journal of Applied Entomology. 2014;51(1): 13–25. 10.7679/j.issn.2095-1353.2014.002

[4] Matsuda K, Buckingham SD, Kleier D, Rauh JJ, Grauso M, Sattelle DB. Neonicotinoids: insecticides acting on insect nicotinic acetylcholine receptors. Trends in Pharmacological Sciences. 2001;22(11): 573–580. 10.1016/S0165-6147(00)01820-4. . 11698101

[5] Liu ZW, Yao XM, Zhang YX. Insect nicotinic acetylcholine receptors (nAChRs): Important amino acid residues contributing to neonicotinoid insecticides selectivity and resistance. African Journal of Biotechnology. 2008;7: 4935–4939.

[6] Tomizawa M, Casida JE. Neonicotinoid insecticide toxicology: Mechanisms of selective action. Annual Review of Pharmacology and Toxicology. 2005;45: 247–268. . 15822177 10.1146/annurev.pharmtox.45.120403.095930

[7] Zhai Q. Some aspects of progress in insect molecular biology: Molecular mechanisms of insecticide resistance. Acta Entomologica Sinica. 1995;38(4): 493–501.

[8] Ranson H, Claudianos C, Ortelli F, Abgrall C, Hemingway J, Sharakhova MV, et al Evolution of supergene families associated with insecticide resistance. Science. 2002;298(5591): 179–181. 10.1126/science.1076781 . 12364796

[9] Casida JE, Durkin KA. Neuroactive Insecticides: Targets, Selectivity, Resistance, and Secondary Effects. Annual Review of Entomology. 2013;58: 99–117. 10.1146/annurev-ento-120811-153645 . 23317040

[10] Meng XK, Zhang YX, Guo BN, Sun HH, Liu CJ, Liu ZW. Identification of key amino acid differences contributing to neonicotinoid sensitivity between two nAChR α subunits from *Pardosa pseudoannulata* . Neuroscience Letters. 2015;584: 123–128. 10.1016/j.neulet.2014.10.013 . 25459289

[11] Tijet N, Helvig C, Feyereisen R. The cytochrome P450 gene superfamily in Drosophila melanogaster: Annotation, intron-exon organization and phylogeny. Gene. 2001;262(1–2): 189–198. 10.1016/S0378-1119(00)00533-3. . 11179683

[12] Daborn PJ, Lumb C, Boey A, Wong W, Ffrench-Constant RH, Batterham P. Evaluating the insecticide resistance potential of eight *Drosophila melanogaster* cytochrome P450 genes by transgenic over-expression. Insect Biochemistry and Molecular Biology. 2007;37(5): 512–519. 10.1016/j.ibmb.2007.02.008. . 17456446

[13] Zimmer CT, Nauen R. Cytochrome P450 mediated pyrethroid resistance in European populations of *Meligethes aeneus* (Coleoptera: Nitidulidae). Pesticide Biochemistry and Physiology. 2011;100(3): 264–272. 10.1016/j.pestbp.2011.04.011.

[14] Zimmer CT, Bass C, Williamson MS, Kaussmann M, Wölfel K, Gutbrod O, et al Molecular and functional characterization of CYP6BQ23, a cytochrome P450 conferring resistance to pyrethroids in European populations of pollen beetle, *Meligethes aeneus* . Insect Biochemistry and Molecular Biology. 2014;45(0): 18–29. 10.1016/j.ibmb.2013.11.008. . 24316412

[15] Yang X, Xie W, Wang SL, Wu QJ, Pan HP, Li RM, et al Two cytochrome P450 genes are involved in imidacloprid resistance in field populations of the whitefly, *Bemisia tabaci*, in China. Pesticide Biochemistry and Physiology. 2013;107(3): 343–350. 10.1016/j.pestbp.2013.10.002. 10.1016/j.pestbp.2013.10.002 24267696

[16] Riga M, Tsakireli D, Ilias A, Morou E, Myridakis A, Stephanou EG, et al Abamectin is metabolized by CYP392A16, a cytochrome P450 associated with high levels of acaricide resistance in *Tetranychus urticae* . Insect Biochemistry and Molecular Biology. 2014;46(0): 43–53. 10.1016/j.ibmb.2014.01.006. . 24463358

[17] Rasool A, Joußen N, Lorenz S, Ellinger R, Schneider B, Khan SA, et al An independent occurrence of the chimeric P450 enzyme CYP337B3 of *Helicoverpa armigera* confers cypermethrin resistance in Pakistan. Insect Biochemistry and Molecular Biology. 2014;53(0): 54–65. 10.1016/j.ibmb.2014.07.006. . 25064010

[18] Han H, Yu Q, Vila-Aiub M, Powles SB. Genetic inheritance of cytochrome P450-mediated metabolic resistance to chlorsulfuron in a multiple herbicide resistant *Lolium rigidum* population. Crop Protection. 2014;65(0): 57–63. 10.1016/j.cropro.2014.06.026.

[19] Feng LF, Fu CJ, Yuan DX, Miao W. A P450 gene associated with robust resistance to DDT in ciliated protozoan, *Tetrahymena thermophila* by efficient degradation. Aquatic Toxicology. 2014;149(0): 126–132. 10.1016/j.aquatox.2014.02.004. . 24607688

[20] Pavlidi N, Monastirioti M, Daborn P, Livadaras I, Van Leeuwen T, Vontas J. Transgenic expression of the Aedes aegypti CYP9J28 confers pyrethroid resistance in *Drosophila melanogaster* . Pesticide Biochemistry and Physiology. 2012;104(2): 132–135. 10.1016/j.pestbp.2012.07.003.

[21] Ding ZP, Wen YC, Yang BJ, Zhang YX, Liu SH, Liu ZW, et al Biochemical mechanisms of imidacloprid resistance in *Nilaparvata lugens*: Over-expression of cytochrome P450 CYP6AY1. Insect Biochemistry and Molecular Biology. 2013;43(11): 1021–1027. 10.1016/j.ibmb.2013.08.005. 10.1016/j.ibmb.2013.08.005 23994173

[22] Claudianos C, Ranson H, Johnson RM, Biswas S, Schuler MA, Berenbaum MR, et al A deficit of detoxification enzymes: pesticide sensitivity and environmental response in the honeybee. Insect Mol Biol. 2006;15(5): 615–636. 10.1111/j.1365-2583.2006.00672.x . 17069637 PMC1761136

[23] David JP, Strode C, Vontas J, Nikou D, Vaughan A, Pignatelli PM, et al The *Anopheles gambiae* detoxification chip: a highly specific microarray to study metabolic-based insecticide resistance in malaria vectors. Proc Natl Acad Sci USA. 2005;102(11): 4080–4084. 10.1073/pnas.0409348102 . 15753317 PMC554807

[24] Pridgeon JW, Zhang L, Liu NN. Overexpression of CYP4G19 associated with a pyrethroid-resistant strain of the German cockroach, *Blattella germanica* (L.). Gene. 2003;314: 157–163. 10.1016/s0378-1119(03)00725-x . 14527728

[25] Feyereisen R. Evolution of insect P450. Biochemical Society Transactions. 2006;34: 1252–1255. 17073796 10.1042/BST0341252

[26] Wei DD, Chen EH, Ding TB, Chen SC, Dou W, Wang JJ. De novo assembly, gene annotation, and marker discovery in stored-product pest *Liposcelis entomophila* (Enderlein) using transcriptome sequences. Plos One. 2013;8(11): e80046 10.1371/journal.pone.0080046 . 24244605 PMC3828239

[27] Karatolos N, Pauchet Y, Wilkinson P, Chauhan R, Denholm I, Gorman K, et al Pyrosequencing the transcriptome of the greenhouse whitefly, *Trialeurodes vaporariorum* reveals multiple transcripts encoding insecticide targets and detoxifying enzymes. Bmc Genomics. 2011;12: 56 10.1186/1471-2164-12-56 . 21261962 PMC3036619

[28] Oakeshott JG, Johnson RM, Berenbaum MR, Ranson H, Cristino AS, Claudianos C. Metabolic enzymes associated with xenobiotic and chemosensory responses in *Nasonia vitripennis* . Insect Mol Biol. 2010;19 Suppl 1: 147–163. 10.1111/j.1365-2583.2009.00961.x . 20167025

[29] Lee SH, Kang JS, Min JS, Yoon KS, Strycharz JP, Johnson R, et al Decreased detoxification genes and genome size make the human body louse an efficient model to study xenobiotic metabolism. Insect Mol Biol. 2010;19(5): 599–615. 10.1111/j.1365-2583.2010.01024.x . 20561088 PMC2944910

[30] Pavlidi N, Dermauw W, Rombauts S, Chrisargiris A, Van T, Leeuwen, et al Analysis of the olive fruit *FlyBactrocera oleae* transcriptome and phylogenetic classification of the major detoxification gene families. Plos One. 2013;8(6): e66533 10.1371/journal.pone.0066533.t001 . 23824998 PMC3688913

[31] Deng H, Huang Y, Feng Q, Zheng S. Two epsilon glutathione *S* -transferase cDNAs from the common cutworm, *Spodoptera litura*: Characterization and developmental and induced expression by insecticides. Journal of Insect Physiology. 2009;55(12): 1174–1183. 10.1016/j.jinsphys.2009.08.017. 10.1016/j.jinsphys.2009.08.017 19715699

[32] Huang HS, Hu NT, Yao YE, Wu CY, Chiang SW, Sun CN. Molecular cloning and heterologous expression of a glutathione S-transferase involved in insecticide resistance from the diamondback moth, Plutella xylostella. Insect Biochemistry and Molecular Biology. 1998;28(9): 651–658. 10.1016/S0965-1748(98)00049-6. . 9755475

[33] Vontas JG, Small G.J., Hemingway J. Glutathione *S*-transferases as antioxidant defence agents confer pyrethroid resistance in *Nilaparvata lugens* . Biochemical Journal. 2001;357: 65–72. . 11415437 10.1042/0264-6021:3570065 PMC1221929

[34] Xu ZF, Zhu WY, Liu YC, Liu X, Chen QX, Peng M, et al Analysis of insecticide resistance-related genes of the Carmine spider mite *Tetranychus cinnabarinus* based on a de novo assembled transcriptome. Plos One. 2014;9(5): e94779 10.1371/journal.pone.0094779 . 24830288 PMC4022505

[35] Zhang YX, Shao Y, Jiang F, Li J, Liu ZW. Identification of two acetylcholinesterases in *Pardosa pseudoannulata* and the sensitivity to insecticides. Insect Biochemistry and Molecular Biology. 2014;46(0): 25–30. 10.1016/j.ibmb.2014.01.004. . 24463359

[36] Villatte F, Ziliani P, Marcel V, Menozzi P, Fournier D. A high number of mutations in insect acetylcholinesterase may provide insecticide resistance. Pesticide Biochemistry and Physiology. 2000;67(2): 95–102. 10.1006/pest.2000.2478

[37] Malekmohammadi M, Hejazi MJ, Mossadegh MS, Galehdari H, Khanjani M, Goodarzi MT. Molecular diagnostic for detecting the acetylcholinesterase mutations in insecticide-resistant populations of Colorado potato beetle, *Leptinotarsa decemlineata* (Say). Pesticide Biochemistry and Physiology. 2012;104(2): 150–6. 10.1016/j.pestbp.2012.06.004.

[38] Kwon DH, Cha DJ, Kim YH, Lee SW, Lee SH. Cloning of the acetylcholinesterase 1 gene and identification of point mutations putatively associated with carbofuran resistance in *Nilaparvata lugens* . Pesticide Biochemistry and Physiology. 2012;103(2): 94–100. 10.1016/j.pestbp.2012.04.003.

[39] Kim JI, Joo YR, Kwon M, Kim GH, Lee SH. Mutation in ace1 associated with an insecticide resistant population of *Plutella xylostella* . Journal of Asia-Pacific Entomology. 2012;15(3): 401–407. 10.1016/j.aspen.2012.02.008.

[40] Liu ZW, Williamson MS, Lansdell SJ, Denholm I, Han ZJ, Millar NS. A nicotinic acetylcholine receptor mutation conferring target-site resistance to imidacloprid in *Nilaparvata lugens* (brown planthopper). Proc Natl Acad Sci USA. 2005;102(24): 8420–8425. 10.1073/pnas.0502901102 . 15937112 PMC1150837

[41] Puinean AM, Lansdell SJ, Collins T, Bielza P, Millar NS. A nicotinic acetylcholine receptor transmembrane point mutation (G275E) associated with resistance to spinosad in *Frankliniella occidentalis* . Journal of Neurochemistry. 2013;124(5): 590–601. 10.1111/jnc.12029 . 23016960 PMC3644170

[42] Puinean AM, Elias J, Slater R, Warren A, Field LM, Williamson MS, et al Development of a high-throughput real-time PCR assay for the detection of the R81T mutation in the nicotinic acetylcholine receptor of neonicotinoid-resistant *Myzus persicae* . Pest Management Science. 2013;69(2): 195–199. 10.1002/ps.3300 . 22528746

[43] Shi XG, Zhu YK, Xia XM, Qiao K, Wang HY, Wang KY. The mutation in nicotinic acetylcholine receptor beta 1 subunit may confer resistance to imidacloprid in *Aphis gossypii* (Glover). Journal of Food Agriculture & Environment. 2012;10(2): 1227–1230.

[44] Bass C, Puinean AM, Andrews M, Cutler P, Daniels M, Elias J, et al Mutation of a nicotinic acetylcholine receptor beta subunit is associated with resistance to neonicotinoid insecticides in the aphid *Myzus persicae* . BMC Neuroscience. 2011;12: 51 10.1186/1471-2202-12-51 . 21627790 PMC3121619

[45] Song F, You ZQ, Yao XM, Cheng JG, Liu ZW, Lin KJ. Specific loops D, E and F of nicotinic acetylcholine receptor beta1 subunit may confer imidacloprid selectivity between *Myzus persicae* and its predatory enemy *Pardosa pseudoannulata* . Insect Biochemistry and Molecular Biology. 2009;39(11): 833–841. 10.1016/j.ibmb.2009.09.009 . 19818849

[46] Zheng Y, Priest B, Cully DF, Ludmerer SW. RdlDv, a novel GABA-gated chloride channel gene from the American dog tick *Dermacentor variabilis* . Insect Biochemistry and Molecular Biology. 2003;33(6): 595–599. 10.1016/s0965-1748(03)00038-9 12770577

[47] Domingues LN, Guerrero FD, Becker ME, Alison MW, Foil LD. Discovery of the Rdl mutation in association with a cyclodiene resistant population of horn flies, *Haematobia irritans* (Diptera: Muscidae). Vet Parasitol. 2013;198(1–2): 172–179. 10.1016/j.vetpar.2013.08.023 . 24055107

[48] Wondji CS, Dabire RK, Tukur Z, Irving H, Djouaka R, Morgan JC. Identification and distribution of a GABA receptor mutation conferring dieldrin resistance in the malaria vector *Anopheles funestus* in Africa. Insect Biochemistry and Molecular Biology. 2011;41(7): 484–491. 10.1016/j.ibmb.2011.03.012 . 21501685 PMC3579012

[49] Nakao T, Naoi A, Kawahara N, Hirase K. Mutation of the GABA receptor associated with fipronil resistance in the whitebacked planthopper, *Sogatella furcifera* . Pesticide Biochemistry and Physiology. 2010;97(3): 262–266. 10.1016/j.pestbp.2010.03.006

[50] Kehoe J, Buldakova S, Acher F, Dent J, Bregestovski P, Bradley J. Aplysia cy*S*-loop glutamate-gated chloride channels reveal convergent evolution of ligand specificity. Journal of Molecular Evolution. 2009;69(2): 125–41. 10.1007/s00239-009-9256-z . 19554247

[51] Kita T, Ozoe F, Ozoe Y. Expression pattern and function of alternative splice variants of glutamate-gated chloride channel in the housefly *Musca domestica* . Insect Biochemistry and Molecular Biology. 2014;45: 1–10. 10.1016/j.ibmb.2013.11.004 . 24291284

[52] Kwon DH, Yoon KS, Clark JM, Lee SH. A point mutation in a glutamate-gated chloride channel confers abamectin resistance in the two-spotted spider mite, *Tetranychus urticae* Koch. Insect Mol Biol. 2010;19(4): 583–591. 10.1111/j.1365-2583.2010.01017.x . 20522121

[53] Grabherr MG, Haas BJ, Yassour M, Levin JZ, Thompson DA, Amit I, et al Full-length transcriptome assembly from RNA-Seq data without a reference genome. Nature Biotechnology. 2011;29(7): 644–U130. 10.1038/nbt.1883 . 21572440 PMC3571712

[54] Conesa A, Gotz S, Garcia-Gomez JM, Terol J, Talon M, Robles M. Blast2GO: a universal tool for annotation, visualization and analysis in functional genomics research. Bioinformatics. 2005;21(18): 3674–3676. 10.1093/bioinformatics/bti610 . 16081474

[55] Ashburner M, Ball CA, Blake JA, Botstein D, Butler H, Cherry JM, et al Gene Ontology: tool for the unification of biology. Nature Genetics. 2000;25(1): 25–29. . 10802651 10.1038/75556 PMC3037419

[56] Thompson JD, Gibson TJ, Plewniak F, Jeanmougin F, Higgins DG. The CLUSTAL_X windows interface: flexible strategies for multiple sequence alignment aided by quality analysis tools. Nucleic Acids Research. 1997;25(24): 4876–4882. 10.1093/nar/25.24.4876 . 9396791 PMC147148

[57] Tamura K, Peterson D, Peterson N, Stecher G, Nei M, Kumar S. MEGA5: molecular evolutionary genetics analysis using maximum likelihood, evolutionary distance, and maximum parsimony methods. Mol Biol Evol. 2011;28(10): 2731–2739. doi: 10.1093/molbev/msr121. 21546353 21546353 10.1093/molbev/msr121 PMC3203626

