# Supplementary figures and images for: Sequence Analysis of Insecticide Action and Detoxification-Related Genes in the Insect Pest Natural Enemy Pardosa pseudoannulata
Source: PLoS One. 2015 Apr 29;10(4):e0125242. doi: 10.1371/journal.pone.0125242 (PMC4414451; doi:10.1371/journal.pone.0125242)

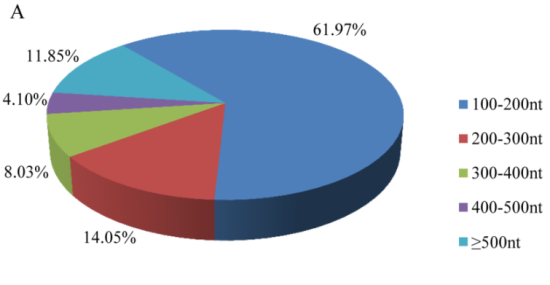

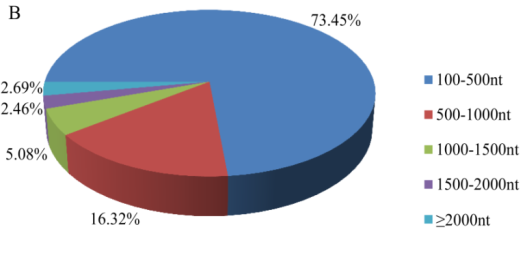


**S1 Fig. Length distribution of *P. pseudoannulata* transcriptome sequences.**

Supplement: S1 Fig — (DOCX) [file pone.0125242.s001.docx]
