# Supplementary material for: Sequence Analysis of Insecticide Action and Detoxification-Related Genes in the Insect Pest Natural Enemy Pardosa pseudoannulata
Source: PLoS One. 2015 Apr 29;10(4):e0125242. doi: 10.1371/journal.pone.0125242 (PMC4414451; doi:10.1371/journal.pone.0125242)
Supplement: S2 Fig — (DOCX) [file pone.0125242.s002.docx]

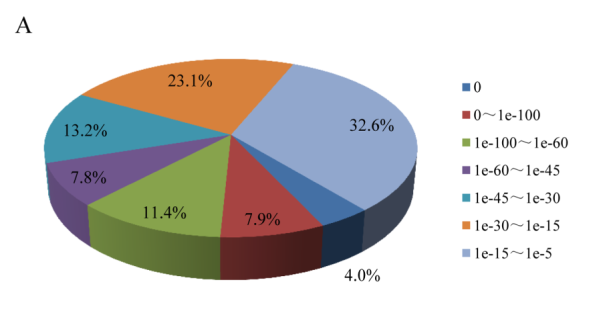

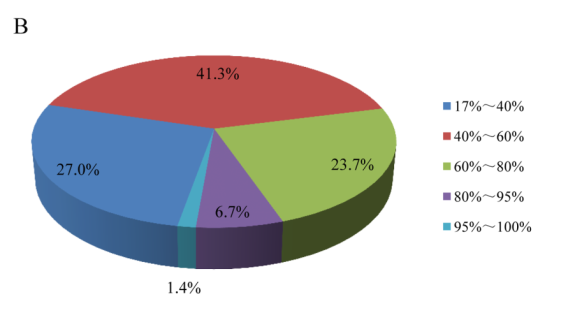


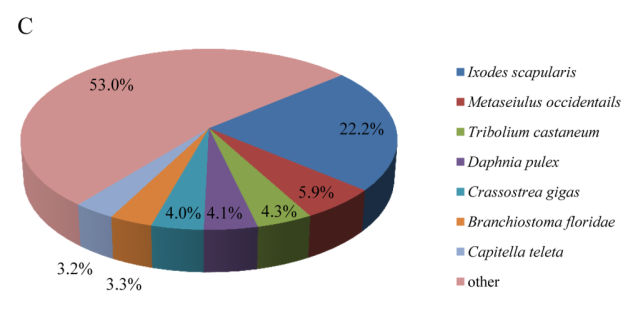


**S2 Fig. Homology analysis of unigenes for *P. pseudoannulata*.** A: E-value distribution of BLAST hits for each unigenes with a cut-off E-value of 1.0E^-5^. B: Similarity distribution of the top BLAST hits for each sequence. C: Species distribution.
