# Supplementary material for: Sequence Analysis of Insecticide Action and Detoxification-Related Genes in the Insect Pest Natural Enemy Pardosa pseudoannulata
Source: PLoS One. 2015 Apr 29;10(4):e0125242. doi: 10.1371/journal.pone.0125242 (PMC4414451; doi:10.1371/journal.pone.0125242)
Supplement: S3 Fig — (DOCX) [file pone.0125242.s003.docx]

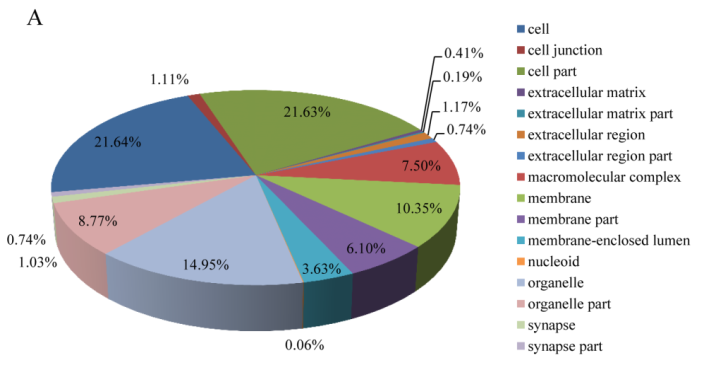

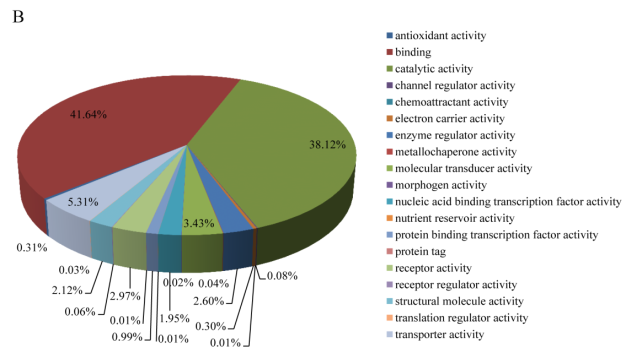

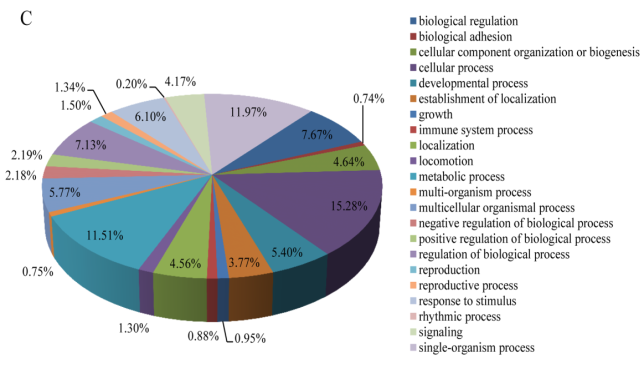


**S3 Fig. Gene Ontology (GO) annotation and classification of the *P. pseudoannulata* transcriptome.** A: Cellular component. B: Molecular function. C: Biological process.
