# Supplementary material for: Sequence Analysis of Insecticide Action and Detoxification-Related Genes in the Insect Pest Natural Enemy Pardosa pseudoannulata
Source: PLoS One. 2015 Apr 29;10(4):e0125242. doi: 10.1371/journal.pone.0125242 (PMC4414451; doi:10.1371/journal.pone.0125242)
Supplement: S5 Fig — (DOCX) [file pone.0125242.s005.docx]

**A**


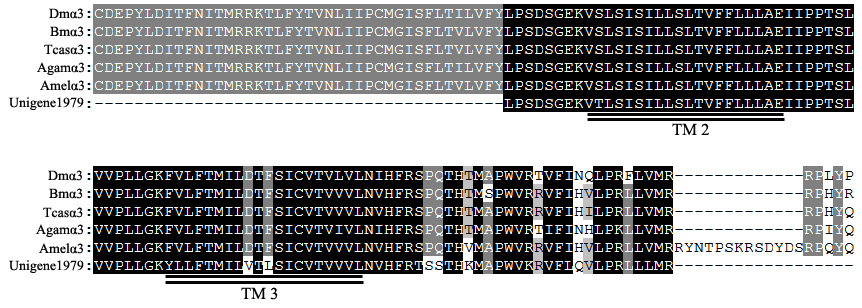


**B**


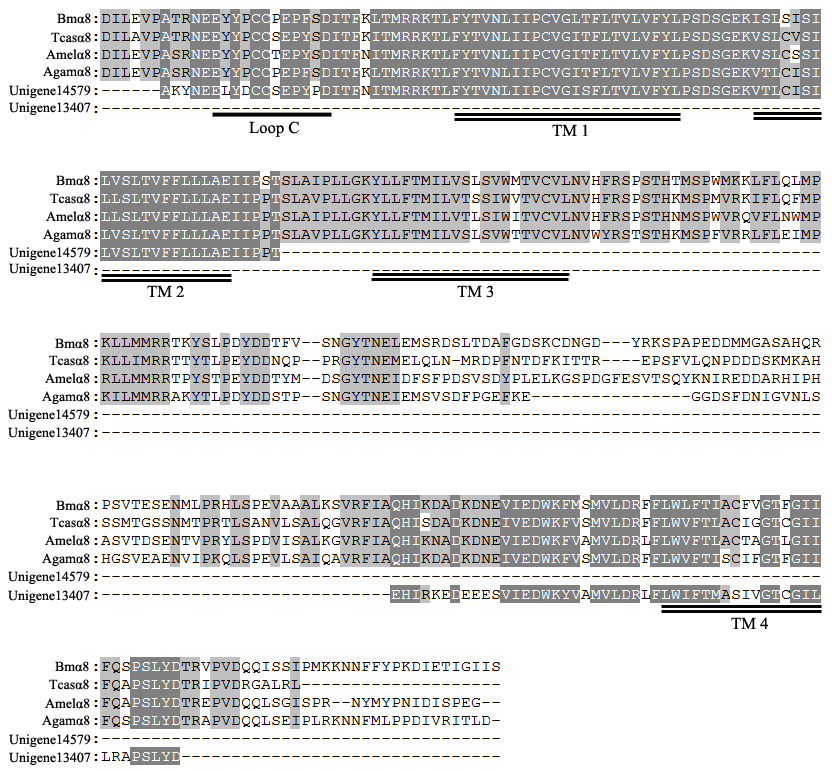


**C**


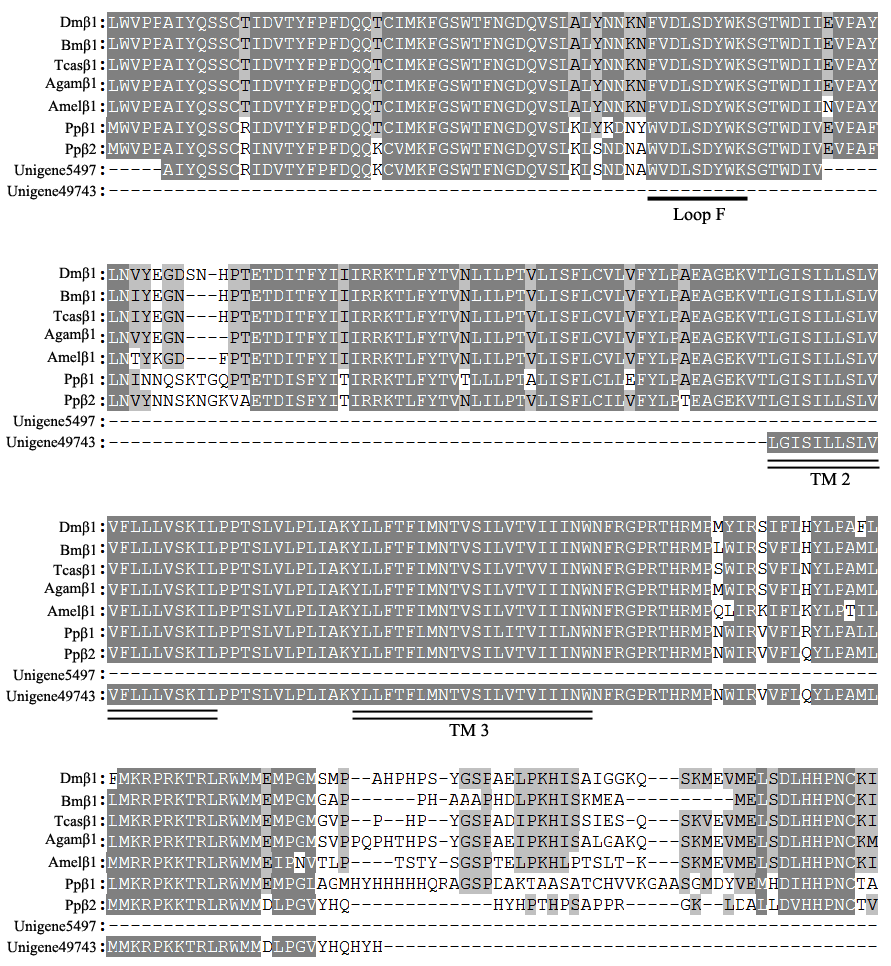


**D**


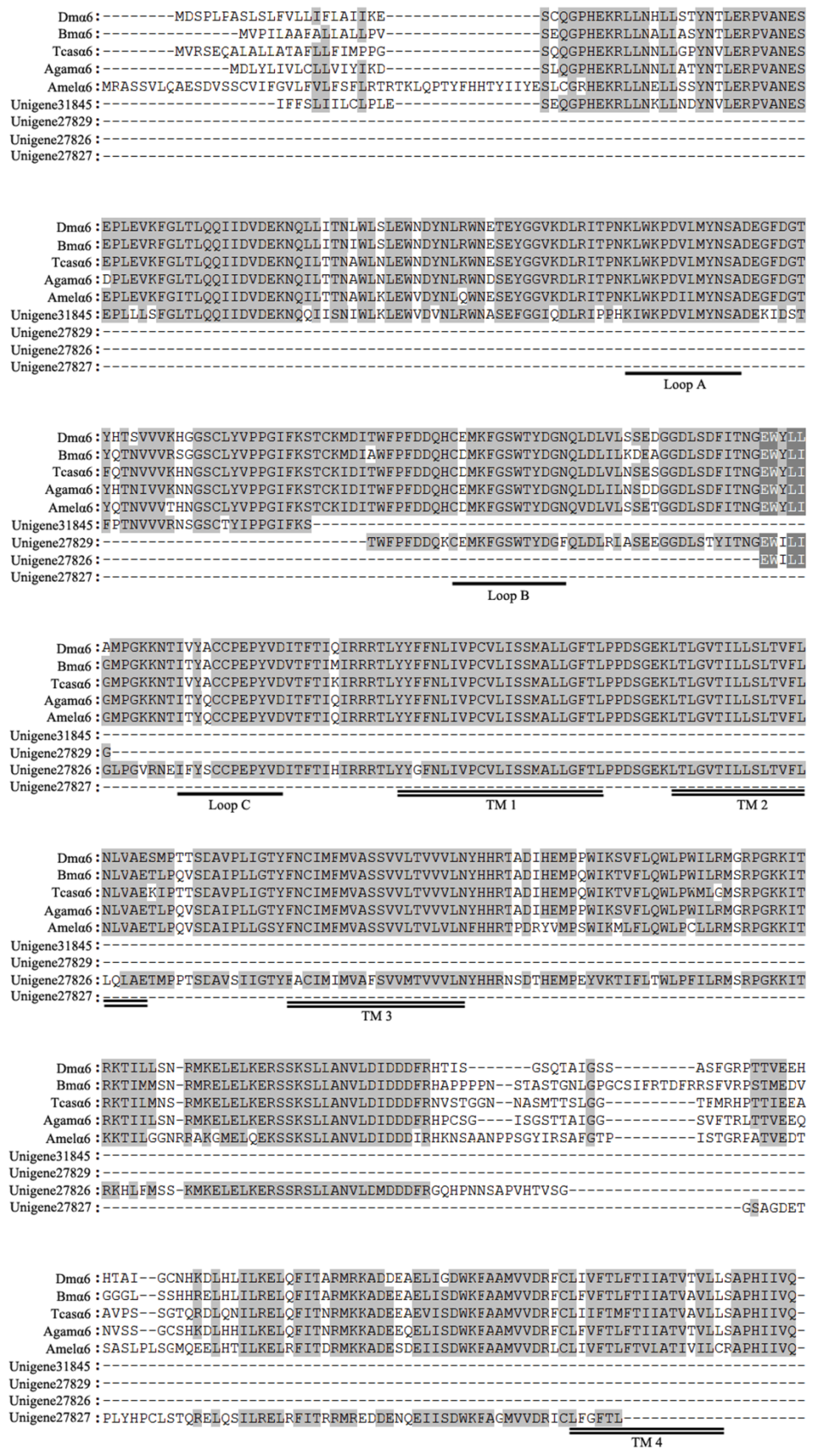


**S5 Fig. Alignment of insect nAChR subunits with unigenes in the *P. pseudoannulata* transcriptome.** Loops A, B,C and F, important to agonist binding in nAChRs subunit, underlined by single line. Transmembrane domains (TM 1-4) are marked by double lines. A, Dm: *Drosophila melanogaster* (CAA75688); Bm: *Bombyx mori* (ABV72685); Tcas: *Tribolium castaneum* (EEZ97689); Aga: *Anopheles gambiae* (AAU12505); Amel: *Apis mellifera* (AAY87891). B, Bm: *Bombyx mori* (ABV72690); Tcas: *Tribolium castaneum* (EEZ99341); Amel: *Apis mellifera* (NP_001011575); Aga: *Anopheles gambiae* (AAU12512). C, Dm: *Drosophila melanogaster* (AAF47900); Bm: *Bombyx mori* (ABV72692); Tcas: *Tribolium castaneum* (EFA12056); Aga: *Anopheles gambiae* (AAU12514); Amel: *Apis mellifera* (AAY87897); Pp: *Pardosa pseudoannulata* (Ppβ1, ACT35385; Ppβ2, ADG63463). D, Dm: *Drosophila melanogaster* (NP_723494); Bm: *Bombyx mori* (NP_001091842); Tcas: *Tribolium castaneum* (EFA11604); Aga: *Anopheles gambiae* (AAU12509); Amel: *Apis mellifera* (NP_001073564).
