# Supplementary material for: Sequence Analysis of Insecticide Action and Detoxification-Related Genes in the Insect Pest Natural Enemy Pardosa pseudoannulata
Source: PLoS One. 2015 Apr 29;10(4):e0125242. doi: 10.1371/journal.pone.0125242 (PMC4414451; doi:10.1371/journal.pone.0125242)
Supplement: S6 Fig — (DOCX) [file pone.0125242.s006.docx]

**A**


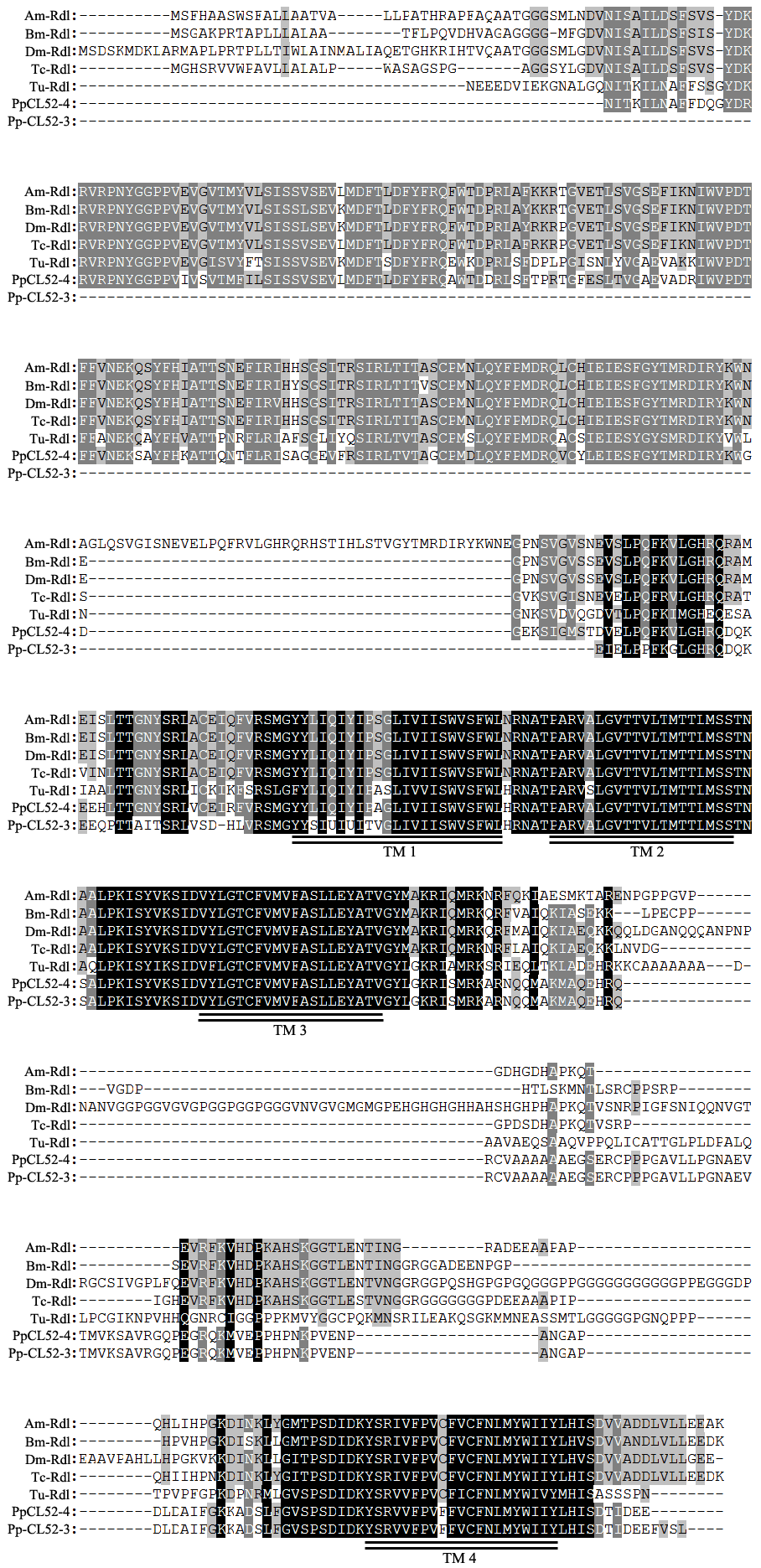


**B**


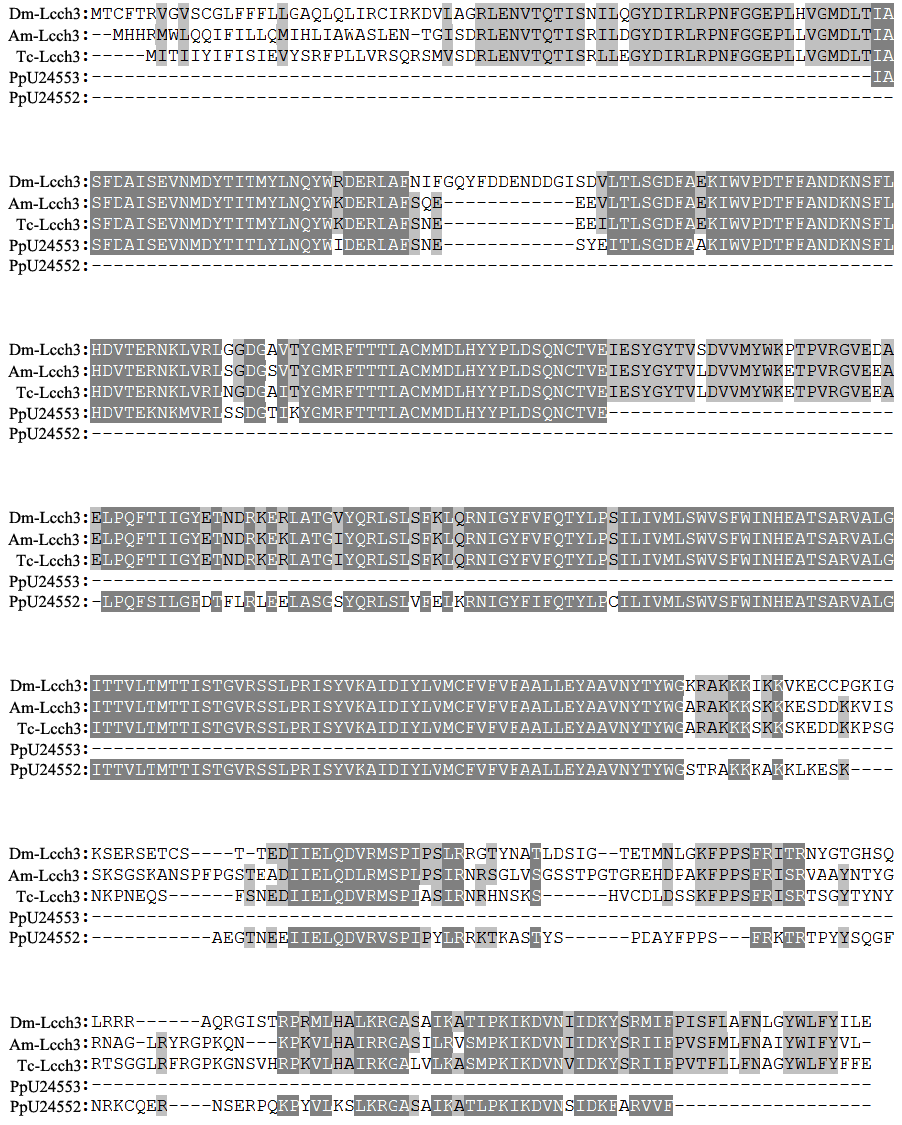


**C**


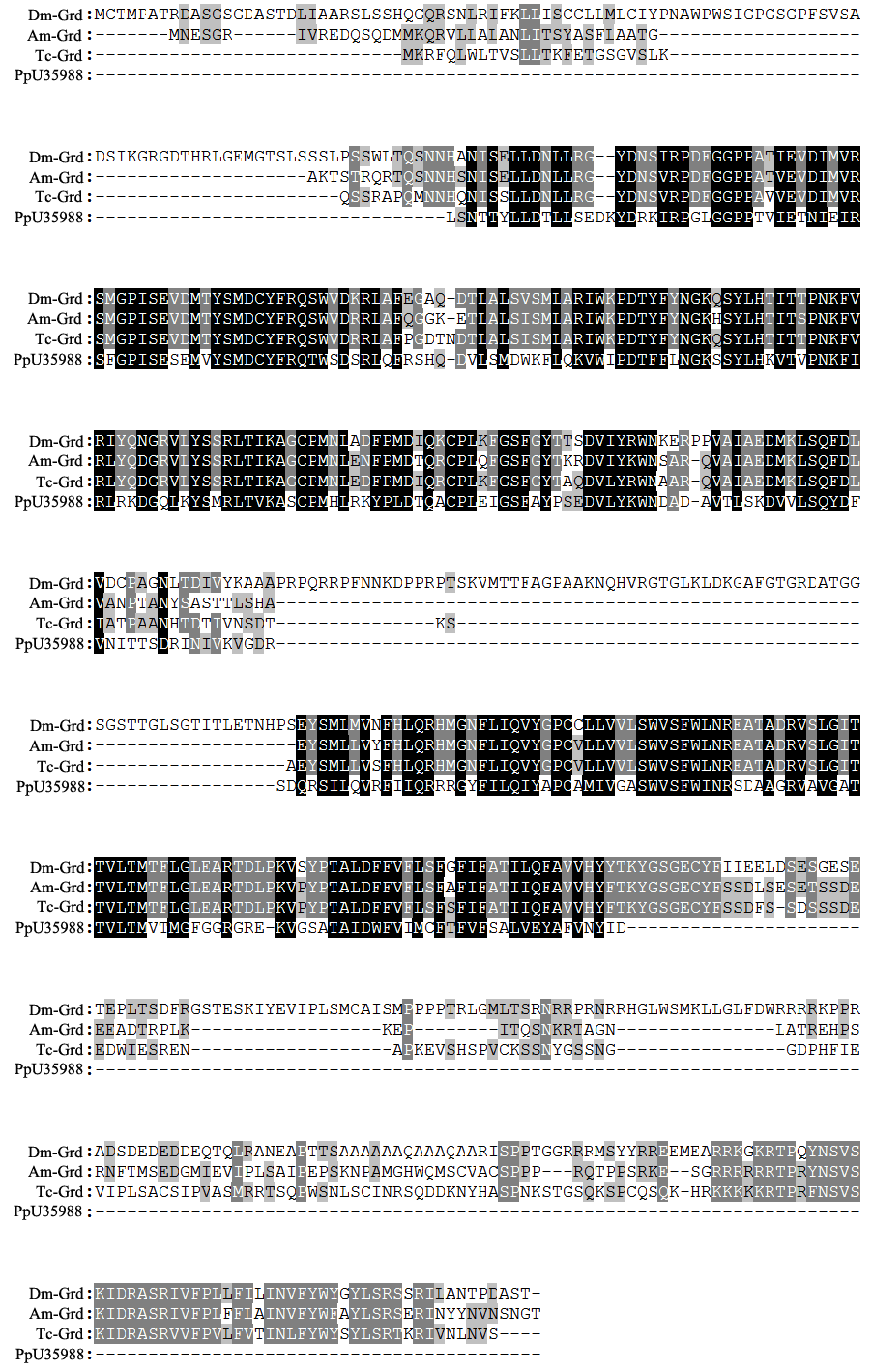


**S6 Fig. Alignment of species GABA receptors, Rdl, Lcch3, and Grd, with unigenes in the *P. pseudoannulata* transcriptome.** A: Transmembrane regions (T1–T4) were marked by double line. Am: *Apis mellifera* (XP_001120292); Bm: *Bombyx mori* (NP_001093294); Dm: *Drosophila melanogaster* (NP_523991); Tc: *Tribolium castaneum* (NP_001107808); Tu: *Tetranychus urticae* (AFG29912). B: Dm: *Drosophila melanogaster* (NP_996469); Am: *Apis mellifera* (NP_001071280); Tc: *Tribolium castaneum* (NP_001103251). C: Dm: *Drosophila melanogaster* (NP_524131); Am: *Apis mellifera* (XP_006563295); Tc: *Tribolium castaneum* (NP_001107772).
