# Supplementary material for: Sequence Analysis of Insecticide Action and Detoxification-Related Genes in the Insect Pest Natural Enemy Pardosa pseudoannulata
Source: PLoS One. 2015 Apr 29;10(4):e0125242. doi: 10.1371/journal.pone.0125242 (PMC4414451; doi:10.1371/journal.pone.0125242)
Supplement: S7 Fig — (DOCX) [file pone.0125242.s007.docx]

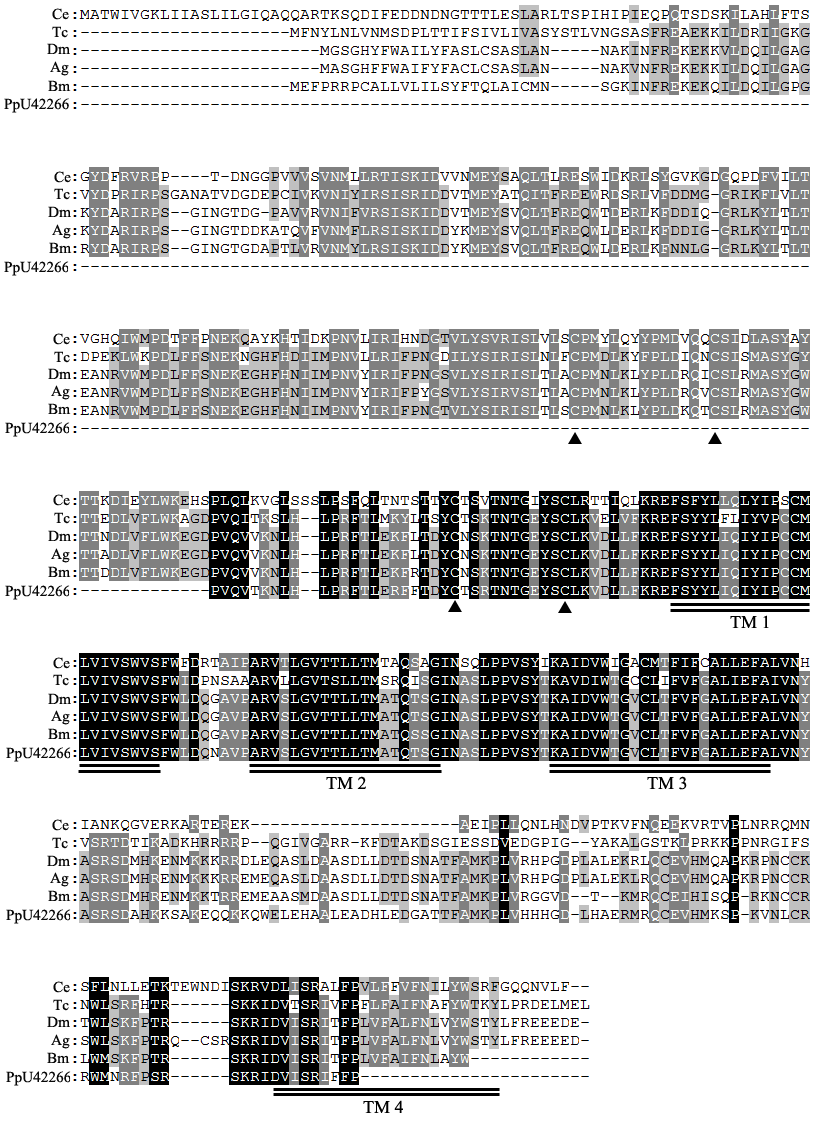


**S7 Fig. Alignment of species glutamate-gated chloride channels with unigenes in the *P. pseudoannulata* transcriptome.** Transmembrane regions (T1–T4) were marked by double line and the signature cysteine residues are indicated by black triangle. Ce: *Caenorhabditis elegans* (AAA50785); Tc: *Tetranychus cinnabarinus* (AGJ03543); Dm: *Drosophila melanogaster* (ABC66182); Ag: *Anopheles gambiae* (AGS43089); Bm: *Bombyx mori* (BAO58781).
