# Supplementary material for: Sequence Analysis of Insecticide Action and Detoxification-Related Genes in the Insect Pest Natural Enemy Pardosa pseudoannulata
Source: PLoS One. 2015 Apr 29;10(4):e0125242. doi: 10.1371/journal.pone.0125242 (PMC4414451; doi:10.1371/journal.pone.0125242)
Supplement: S1 Table — (DOCX) [file pone.0125242.s008.docx]

| **Sequencing** | |
| --- | --- |
| Total number of reads | 80,331,446 |
| Total number of clean reads | 73,119,274 |
| Total clean nucleotides (nt) | 6,580,734,660 |
| Q20 percentage (%) | 97.62% |
| N percentage (%) | 0.00% |
| GC percentage (%) | 43.18% |
| Number of contigs | 124,054 |
| Length of contigs (nt) | 36,056,505 |
| Mean length of contigs (nt) | 291 |
| Number of unigenes | 65,787 |
| Length of unigenes (nt) | 32,438,492 |
| Mean length of unigenes (nt) | 493 |
| Cluster of unigenes | 8,756 |
| Singleton of unigenes | 57,031 |
| Unigenes annotations against Nr | 24,035 |
| Unigenes annotations against Swiss-Prot | 20,358 |
| Unigenes annotations against KEGG | 17,257 |
| Unigenes annotations against COG | 7,805 |
| Unigenes annotations against GO | 13,205 |

**S1 Table.** Summary of the statistics for Illumina sequencing of the *P. pseudoannulata* transcriptome.
