# Supplementary material for: Sequence Analysis of Insecticide Action and Detoxification-Related Genes in the Insect Pest Natural Enemy Pardosa pseudoannulata
Source: PLoS One. 2015 Apr 29;10(4):e0125242. doi: 10.1371/journal.pone.0125242 (PMC4414451; doi:10.1371/journal.pone.0125242)
Supplement: S3 Table — (DOCX) [file pone.0125242.s010.docx]

**S3 Table.** Manually identified P450 unigenes from the *P. pseudoannulata* transcriptome.

| **Gene ID** | **Gene Length** | **Number of reads** | **Nr-Evalue** | **Clade** | **Nr-annotation** |
| --- | --- | --- | --- | --- | --- |
| CL991.Contig1 | 1792 | 1320 | 6.00E-99 | CYP 3 | 3A4 |
| Unigene33119 | 1203 | 706 | 8.00E-68 |  | 3A4 |
| CL2935.Contig2 | 713 | 74 | 7.00E-47 |  | 3A6 |
| CL2935.Contig1 | 255 | 27 | 6.00E-21 |  | 3A7 |
| Unigene22737 | 979 | 130 | 1.00E-59 |  | 3A8 |
| Unigene11800 | 525 | 61 | 9.00E-40 |  | 3A8 |
| CL277.Contig1 | 1692 | 367 | 9.00E-98 |  | 3A8 |
| Unigene40544 | 469 | 48 | 9.00E-17 |  | 3A11 |
| Unigene23255 | 526 | 63 | 1.00E-20 |  | 3A11 |
| Unigene6738 | 152 | 10 | 9.00E-07 |  | 3A12 |
| Unigene32427 | 1822 | 2244 | 9.00E-101 |  | 3A13 |
| Unigene22682 | 479 | 56 | 5.00E-40 |  | 3A14 |
| Unigene16954 | 1659 | 1117 | 7.00E-92 |  | 3A41 |
| CL3120.Contig1 | 476 | 41 | 1.00E-18 |  | 3A56 |
| Unigene6739 | 256 | 16 | 3.00E-13 |  | 6k1 |
| Unigene22680 | 252 | 30 | 1.00E-16 |  | 9E2 |
| Unigene56136 | 150 | 4 | 1.00E-09 |  | 9Z4 |
| CL3185.Contig1 | 772 | 2174 | 1.00E-36 | CYP 2 | 1A |
| Unigene31335 | 290 | 84 | 8.00E-11 |  | 1D1 |
| Unigene30055 | 690 | 196 | 6.00E-19 |  | 2C8 |
| Unigene53583 | 248 | 8 | 2.00E-06 |  | 2C23 |
| Unigene5688 | 255 | 20 | 8.00E-06 |  | 2C48 |
| Unigene55782 | 173 | 5 | 2.00E-11 |  | 2D3 |
| Unigene53410 | 267 | 14 | 5.00E-17 |  | 2G1 |
| Unigene45623 | 348 | 25 | 2.00E-17 |  | 2H2 |
| Unigene11536 | 1031 | 199 | 3.00E-32 |  | 2J2 |
| CL1515.Contig2 | 491 | 19 | 5.00E-20 |  | 2J2 |
| Unigene5668 | 293 | 17 | 4.00E-12 |  | 2J2 |
| CL1515.Contig1 | 344 | 31 | 2.00E-12 |  | 2J2 |
| Unigene31631 | 836 | 371 | 2.00E-16 |  | 2J2 |
| Unigene46592 | 465 | 37 | 3.00E-21 |  | 2J2 |
| CL642.Contig1 | 1885 | 636 | 1.00E-52 |  | 2J2 |
| Unigene31630 | 915 | 802 | 2.00E-29 |  | 2J5 |
| Unigene32846 | 504 | 115 | 2.00E-24 |  | 2J6 |
| Unigene20899 | 437 | 63 | 6.00E-32 |  | 2J6 |
| Unigene32808 | 1705 | 1395 | 5.00E-95 |  | 2J6 |
| Unigene46160 | 509 | 40 | 4.00E-30 |  | 2J6 |
| Unigene7611 | 684 | 131 | 3.00E-42 |  | 2J6 |
| CL2832.Contig2 | 958 | 423 | 8.00E-67 |  | 2J6 |
| Unigene32416 | 1011 | 393 | 1.00E-13 |  | 2N2 |
| Unigene26794 | 501 | 55 | 1.00E-12 |  | 2P2 |
| Unigene26910 | 1139 | 225 | 1.00E-51 |  | 2R1 |
| Unigene42291 | 413 | 54 | 5.00E-23 |  | 2U1 |
| Unigene5848 | 154 | 6 | 9.00E-07 |  | 2U1 |
| Unigene16884 | 578 | 325 | 4.00E-39 |  | 2U1 |
| Unigene31334 | 440 | 660 | 1.00E-18 |  | 17 |
| Unigene32845 | 568 | 253 | 1.00E-26 |  | 18A1 |
| Unigene37866 | 428 | 87 | 4.00E-23 |  | 18A1 |
| Unigene25862 | 422 | 49 | 8.00E-18 |  | 18A1 |
| Unigene43960 | 521 | 49 | 4.00E-38 |  | 307A1 |
| Unigene39906 | 957 | 112 | 1.00E-58 |  | 307A1 |
| Unigene40732 | 557 | 81 | 6.00E-61 | CYP 4 | 4C3 |
| Unigene48107 | 284 | 16 | 4.00E-31 |  | 4C3 |
| Unigene37345 | 641 | 117 | 9.00E-34 |  | 4C3 |
| Unigene23552 | 417 | 69 | 2.00E-27 |  | 4C3 |
| Unigene38175 | 334 | 76 | 1.00E-24 |  | 4C3 |
| Unigene23553 | 156 | 12 | 1.00E-09 |  | 4C3 |
| Unigene4098 | 358 | 22 | 4.00E-57 |  | 4C3 |
| CL127.Contig3 | 262 | 93 | 4.00E-23 |  | 4C3 |
| CL127.Contig2 | 1784 | 746 | 2.00E-130 |  | 4C3 |
| Unigene36771 | 1783 | 429 | 4.00E-124 |  | 4C3 |
| Unigene33055 | 1216 | 227 | 1.00E-119 |  | 4C3 |
| Unigene30825 | 1052 | 257 | 2.00E-52 |  | 4V2 |
| Unigene14701 | 441 | 59 | 1.00E-54 |  | 4V2 |
| Unigene43411 | 396 | 44 | 1.00E-26 |  | 4V2 |
| Unigene13266 | 415 | 48 | 2.00E-10 |  | 4V2 |
| CL127.Contig4 | 267 | 39 | 1.00E-12 |  | 4V2 |
| Unigene42146 | 297 | 42 | 1.00E-21 |  | 4V2 |
| CL127.Contig1 | 173 | 46 | 8.00E-13 |  | 4V2 |
| Unigene4686 | 162 | 14 | 1.00E-13 |  | 4V2 |
| Unigene21191 | 209 | 47 | 6.00E-11 | CYP M | 12A5 |
| Unigene47004 | 273 | 19 | 1.00E-13 |  | 24A1 |
| Unigene12414 | 379 | 56 | 8.00E-19 |  | 24A1 |
| Unigene25158 | 270 | 26 | 4.00E-15 |  | 27B1 |
| Unigene3788 | 474 | 38 | 5.00E-28 |  | 49A1 |
| Unigene42145 | 353 | 32 | 1.00E-17 |  | 49A1 |
| CL60.Contig3 | 342 | 48 | 9.00E-17 |  | 49A1 |
| Unigene27882 | 227 | 15 | 5.00E-12 |  | 49A1 |
| Unigene21190 | 199 | 30 | 9.00E-07 |  | 49A1 |
| Unigene32966 | 800 | 498 | 1.00E-107 | Unclassified |  |
| Unigene25443 | 596 | 142 | 3.00E-24 |  |  |
| Unigene37691 | 588 | 108 | 2.00E-26 |  |  |
| Unigene40513 | 568 | 61 | 7.00E-23 |  |  |
| Unigene14671 | 388 | 63 | 4.00E-25 |  |  |
| Unigene45700 | 235 | 19 | 2.00E-08 |  |  |
| Unigene2526 | 229 | 24 | 3.00E-07 |  |  |
| Unigene12715 | 198 | 27 | 5.00E-07 |  |  |
| Unigene25159 | 196 | 34 | 9.00E-09 |  |  |
| Unigene25157 | 158 | 27 | 2.00E-06 |  |  |
| Unigene56618 | 156 | 7 | 9.00E-07 |  |  |
