# Supplementary material for: Sequence Analysis of Insecticide Action and Detoxification-Related Genes in the Insect Pest Natural Enemy Pardosa pseudoannulata
Source: PLoS One. 2015 Apr 29;10(4):e0125242. doi: 10.1371/journal.pone.0125242 (PMC4414451; doi:10.1371/journal.pone.0125242)
Supplement: S4 Table — (DOCX) [file pone.0125242.s011.docx]

| **Gene ID** | **Gene Length** | **Number of reads** | **Nr-Evalue** | **Classification** |
| --- | --- | --- | --- | --- |
| CL1623.Contig1 | 809 | 358 | 5.00E-63 | delta |
| CL1623.Contig2 | 965 | 305 | 2.00E-62 | delta |
| CL1623.Contig3 | 952 | 1375 | 1.00E-38 | delta |
| Unigene6886 | 774 | 142 | 1.00E-44 | delta |
| Unigene30549 | 789 | 519 | 8.00E-58 | delta |
| Unigene28668 | 791 | 1030 | 1.00E-50 | delta |
| Unigene31710 | 843 | 249 | 4.00E-82 | sigma |
| Unigene9178 | 809 | 483 | 1.00E-35 | sigma |
| Unigene9432 | 473 | 3057 | 3.00E-45 | sigma |
| Unigene35518 | 779 | 822 | 6.00E-61 | sigma |
| Unigene9431 | 803 | 2305 | 3.00E-32 | sigma |
| Unigene31021 | 781 | 587 | 1.00E-63 | microsomal |
| Unigene32509 | 544 | 309 | 3.00E-44 | microsomal |
| Unigene15584 | 384 | 44 | 3.00E-39 | unknown |

**S4 Table**. Manually identified GST unigenes from the *P. pseudoannulata* transcriptome.
