# Supplementary material for: Sequence Analysis of Insecticide Action and Detoxification-Related Genes in the Insect Pest Natural Enemy Pardosa pseudoannulata
Source: PLoS One. 2015 Apr 29;10(4):e0125242. doi: 10.1371/journal.pone.0125242 (PMC4414451; doi:10.1371/journal.pone.0125242)
Supplement: S5 Table — (DOCX) [file pone.0125242.s012.docx]

| **Gene ID** | **Gene Length** | **Number of reads** | **Nr-Evalue** | **Nr-annotation** |
| --- | --- | --- | --- | --- |
| Unigene29249 | 2853 | 522 | 0 | acetylcholinesterase 1 |
| Unigene50127 | 153 | 11 | 2.00E-13 | acetylcholinesterase 2 |
| Unigene287 | 2248 | 1513 | 1.00E-99 | acetylcholinesterase-like |
| CL2866.Contig1 | 1957 | 120 | 4.00E-101 | acetylcholinesterase-like |
| CL4230.Contig1 | 1802 | 23 | 1.00E-90 | acetylcholinesterase-like |
| CL4230.Contig2 | 1790 | 97 | 1.00E-90 | acetylcholinesterase-like |
| CL2866.Contig2 | 1763 | 92 | 4.00E-101 | acetylcholinesterase-like |
| CL3561.Contig1 | 992 | 170 | 6.00E-36 | acetylcholinesterase-like |
| CL2350.Contig1 | 359 | 65 | 1.00E-34 | acetylcholinesterase-like |
| Unigene18962 | 501 | 39 | 6.00E-23 | acetylcholinesterase |
| Unigene19577 | 1225 | 290 | 1.00E-23 | acetylcholinesterase, putative |
| Unigene24543 | 871 | 151 | 6.00E-52 | acetylcholinesterase, putative |
| Unigene15709 | 732 | 160 | 5.00E-53 | acetylcholinesterase, putative |
| Unigene32130 | 727 | 170 | 9.00E-34 | acetylcholinesterase, putative |
| Unigene2755 | 420 | 47 | 3.00E-30 | acetylcholinesterase, putative |
| Unigene24544 | 189 | 20 | 6.00E-13 | acetylcholinesterase, putative |
| Unigene42388 | 395 | 38 | 7.00E-20 | Acetylcholinesterase precursor |

**S5 Table.** Manually identified AChE unigenes from the *P. pseudoannulata* transcriptome.
