# Supplementary material for: Sequence Analysis of Insecticide Action and Detoxification-Related Genes in the Insect Pest Natural Enemy Pardosa pseudoannulata
Source: PLoS One. 2015 Apr 29;10(4):e0125242. doi: 10.1371/journal.pone.0125242 (PMC4414451; doi:10.1371/journal.pone.0125242)
Supplement: S6 Table — (DOCX) [file pone.0125242.s013.docx]

| **Gene ID** | **Gene Length** | **Number of reads** | **Nr-Evalue** | **Nr-annotation** |
| --- | --- | --- | --- | --- |
| Unigene31345 | 296 | 82 | 2.00E-06 | alpha 7 |
| Unigene14510 | 596 | 74 | 7.00E-101 | alpha 2 |
| Unigene1979 | 281 | 32 | 2.00E-39 | alpha 3 |
| Unigene27826 | 632 | 135 | 3.00E-86 | alpha 6 |
| Unigene27827 | 201 | 25 | 7.00E-14 | alpha 6 |
| Unigene31845 | 1104 | 443 | 6.00E-61 | alpha 6 |
| Unigene27829 | 150 | 15 | 1.00E-19 | alpha 6 |
| Unigene14579 | 252 | 40 | 5.00E-42 | alpha 8 |
| Unigene13407 | 153 | 24 | 2.00E-22 | alpha 8 |
| Unigene36768 | 689 | 153 | 5.00E-12 | alpha 9-like |
| Unigene18960 | 357 | 84 | 4.00E-14 | beta subunit |
| CL3062.Contig1 | 965 | 1639 | 5.00E-37 | beta subunit |
| CL3062.Contig2 | 685 | 2639 | 6.00E-31 | beta subunit |
| CL3062.Contig3 | 877 | 7101 | 1.00E-36 | beta subunit |
| Unigene49743 | 318 | 16 | 9.00E-55 | beta 2 |
| Unigene5497 | 184 | 10 | 6.00E-30 | beta 2 |
| Unigene40260 | 378 | 57 | 7.00E-06 | beta 3 |

**S6 Table**. Manually identified nAChR unigenes from the *P. pseudoannulata* transcriptome.
