# Supplementary material for: Sequence Analysis of Insecticide Action and Detoxification-Related Genes in the Insect Pest Natural Enemy Pardosa pseudoannulata
Source: PLoS One. 2015 Apr 29;10(4):e0125242. doi: 10.1371/journal.pone.0125242 (PMC4414451; doi:10.1371/journal.pone.0125242)
Supplement: S7 Table — (DOCX) [file pone.0125242.s014.docx]

**S7 Table**. Manually identified GABA receptor unigenes from the *P. pseudoannulata* transcriptome.

| **Gene ID** | **Gene Length** | **Number of reads** | **Nr-Evalue** | **Nr-annotation** | **Type** |
| --- | --- | --- | --- | --- | --- |
| Unigene51231 | 150 | 9 | 1.00E-06 | gamma-aminobutyric acid A recptor, subunit alpha | Type A |
| CL52.Contig1 | 1943 | 68 | 6.00E-134 | gamma-aminobutyric acid receptor subunit beta | Type A-Rdl |
| CL52.Contig2 | 1215 | 176 | 4.00E-46 | gamma-aminobutyric acid receptor subunit beta | Type A-Rdl |
| CL52.Contig3 | 1258 | 96 | 2.00E-71 | gamma-aminobutyric acid receptor subunit beta | Type A-Rdl |
| CL52.Contig4 | 1986 | 76 | 1.00E-177 | gamma-aminobutyric acid receptor subunit beta | Type A-Rdl |
| Unigene24553 | 331 | 39 | 4.00E-52 | gamma-aminobutyric acid receptor subunit beta | Type A-Lcch3 |
| Unigene55613 | 220 | 7 | 6.00E-34 | gamma-aminobutyric acid receptor subunit beta | Type A-Lcch3 |
| Unigene24552 | 731 | 423 | 2.00E-84 | GABA receptor beta subunit | Type A-Lcch3 |
| Unigene35988 | 1802 | 767 | 3.00E-116 | gamma-aminobutyric acid receptor alpha | Type A- Grd |
| Unigene47028 | 239 | 19 | 2.00E-09 | gamma-aminobutyric acid receptor alpha | Type B- Gabra6 |
| Unigene56358 | 173 | 6 | 2.00E-16 | gamma-aminobutyric acid type B receptor subunit 2 | Type B- Gabra2 |
| Unigene56493 | 193 | 7 | 1.00E-20 | gamma-aminobutyric acid type B receptor subunit 2 | Type B- Gabra2 |
| Unigene906 | 191 | 21 | 1.00E-23 | gamma-aminobutyric acid type B receptor subunit 2 | Type B- Gabra2 |
| Unigene12212 | 559 | 57 | 1.00E-88 | metabotropic gamma-aminobutyric acid receptor | Type B- Gabra1 |
| Unigene26829 | 452 | 72 | 1.00E-26 | metabotropic gamma-aminobutyric acid receptor | Type B- Gabra1 |
| Unigene3111 | 306 | 30 | 2.00E-37 | metabotropic gamma-aminobutyric acid receptor | Type B- Gabra1 |
| Unigene40429 | 390 | 48 | 5.00E-50 | metabotropic GABA-B receptor subtype | Type B- Gabra1 |
