# Supplementary material for: Sequence Analysis of Insecticide Action and Detoxification-Related Genes in the Insect Pest Natural Enemy Pardosa pseudoannulata
Source: PLoS One. 2015 Apr 29;10(4):e0125242. doi: 10.1371/journal.pone.0125242 (PMC4414451; doi:10.1371/journal.pone.0125242)
Supplement: S8 Table — (DOCX) [file pone.0125242.s015.docx]

| **Gene ID** | **Gene Length** | **Number of reads** | **Nr-Evalue** | **Nr-annotation** |
| --- | --- | --- | --- | --- |
| CL3512.Contig2 | 1786 | 115 | 8.00E-75 | glutamate-gated chloride channel |
| CL3512.Contig1 | 1694 | 8 | 8.00E-75 | glutamate-gated chloride channel |
| CL3316.Contig1 | 1590 | 159 | 6.00E-101 | GluCl alpha |
| CL3316.Contig2 | 1471 | 354 | 5.00E-101 | GluCl alpha |
| Unigene26467 | 889 | 161 | 5.00E-62 | glutamate-gated chloride channel |
| Unigene28941 | 868 | 171 | 7.00E-109 | glutamate-gated chloride channel |
| Unigene36472 | 792 | 262 | 2.00E-49 | glutamate-gated chloride channel |
| Unigene42266 | 673 | 62 | 7.00E-109 | glutamate-gated chloride channel |
| Unigene35964 | 614 | 381 | 9.00E-25 | glutamate-gated chloride channel |
| Unigene20238 | 546 | 392 | 3.00E-33 | glutamate-gated chloride channel |
| Unigene46786 | 311 | 28 | 2.00E-37 | glutamate-gated chloride channel |
| Unigene53049 | 221 | 10 | 2.00E-09 | glutamate-gated chloride channel |

**S8 Table**. Manually identified GluCl unigenes from the *P. pseudoannulata* transcriptome.
